# Supplementary material for: Remodelling of Rea1 linker domain drives the removal of assembly factors from pre-ribosomal particles
Source: Nat Commun. 2024 Nov 27;15:10309. doi: 10.1038/s41467-024-54698-w (PMC11603028; doi:10.1038/s41467-024-54698-w)
Supplement: Supplementary file 1 — Supplementary Information [file 41467_2024_54698_MOESM1_ESM.pdf]

Supplementary Information

Remodelling of Rea1 linker domain drives the removal of assembly factors from pre-ribosomal particles

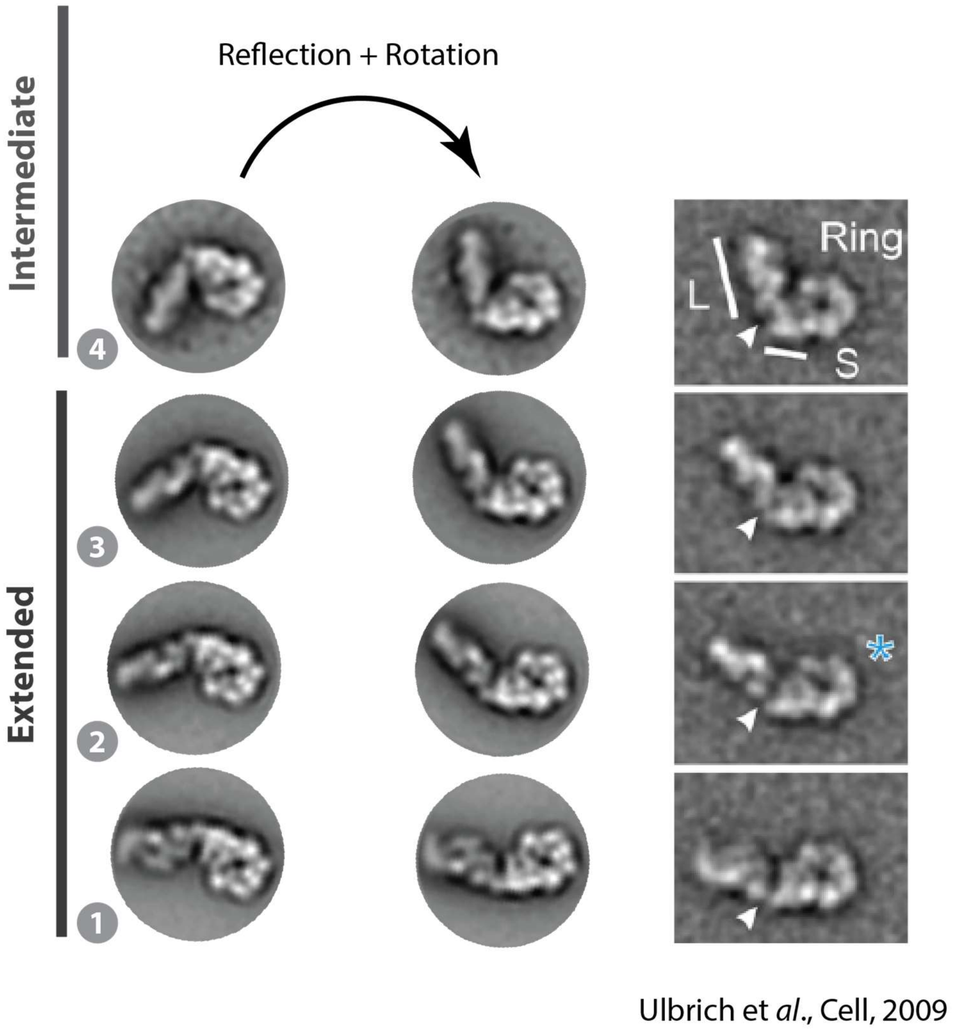

Supplementary Fig. 1: Comparison of Rea1$_{wt}$ negative stain EM 2D classes with published data. States 1 -4 are similar to 2D classes published by Ulbrich et al.

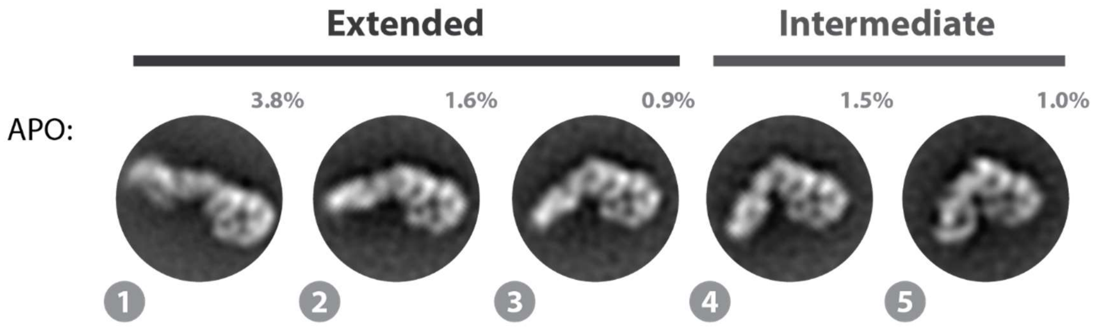

Supplementary Fig. 2: Negative stain EM analysis under APO conditions of EDTA purified Rea1$_{wt}$. Rea1$_{wt}$ was purified in the presence of 5 mM EDTA and subsequently analysed by negative stain EM without adding nucleotide. Only the extended and intermediate linker conformations were detected. Percentage numbers indicate how many particles of the data set sorted into the displayed 2D class averages.

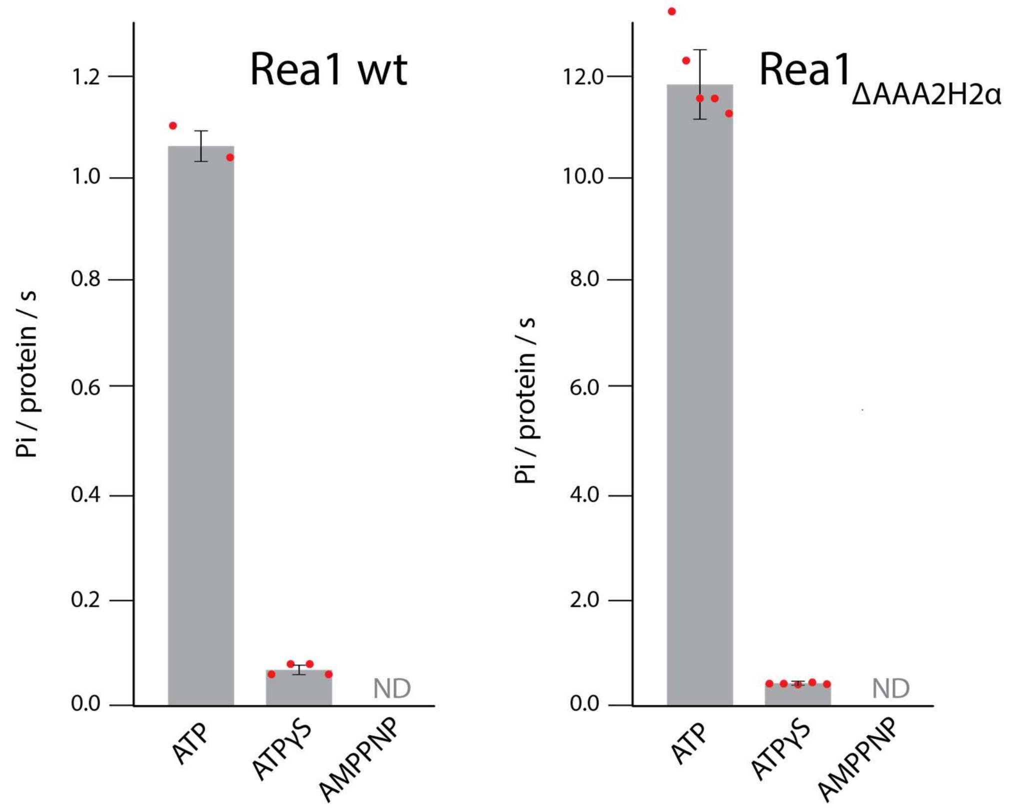

Supplementary Fig. 3: ATPase activities of $Rea1_{wt}$ and $Rea1_{ΔAAA2H2α}$. Right panel: $Rea1_{wt}$ is able to hydrolyse ATP and, at a slower rate, also ATPγS. In the presence of AMPPNP, no $Rea1_{wt}$ hydrolysis activity is detected. Left panel: The $Rea1_{ΔAAA2H2α}$ mutant shows a ≈10 fold higher ATP hydrolysis activity than $Rea1_{wt}$. ATPγS can be hydrolysed at a slower rate. There is no detectable hydrolysis activity with AMPPNP as substrate. $Rea1_{wt}$ + ATP n=2, $Rea1_{wt}$ + ATPγS n=4, $Rea1_{ΔAAA2H2α}$ + ATP n=5, $Rea1_{ΔAAA2H2α}$ + ATPγS n=5. Error bars show the standard deviation.

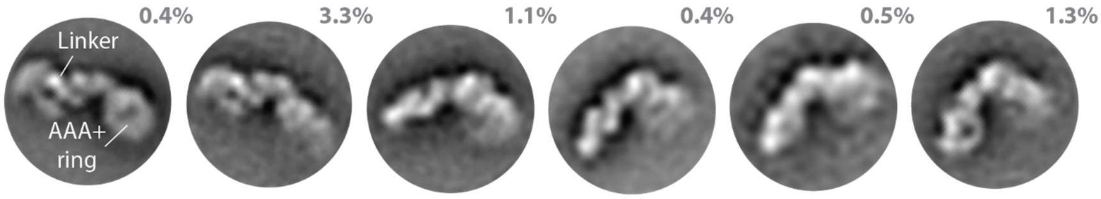

Supplementary Fig. 4: Negative stain 2D class averages of Rea1$_{\Delta AAA2H2\alpha}$ in the absence of nucleotide. Linker conformations consistent with states 1 – 5 of the extended and intermediate classes can be observed. Percentage numbers indicate how many particles of data set sorted into the displayed 2D class averages.

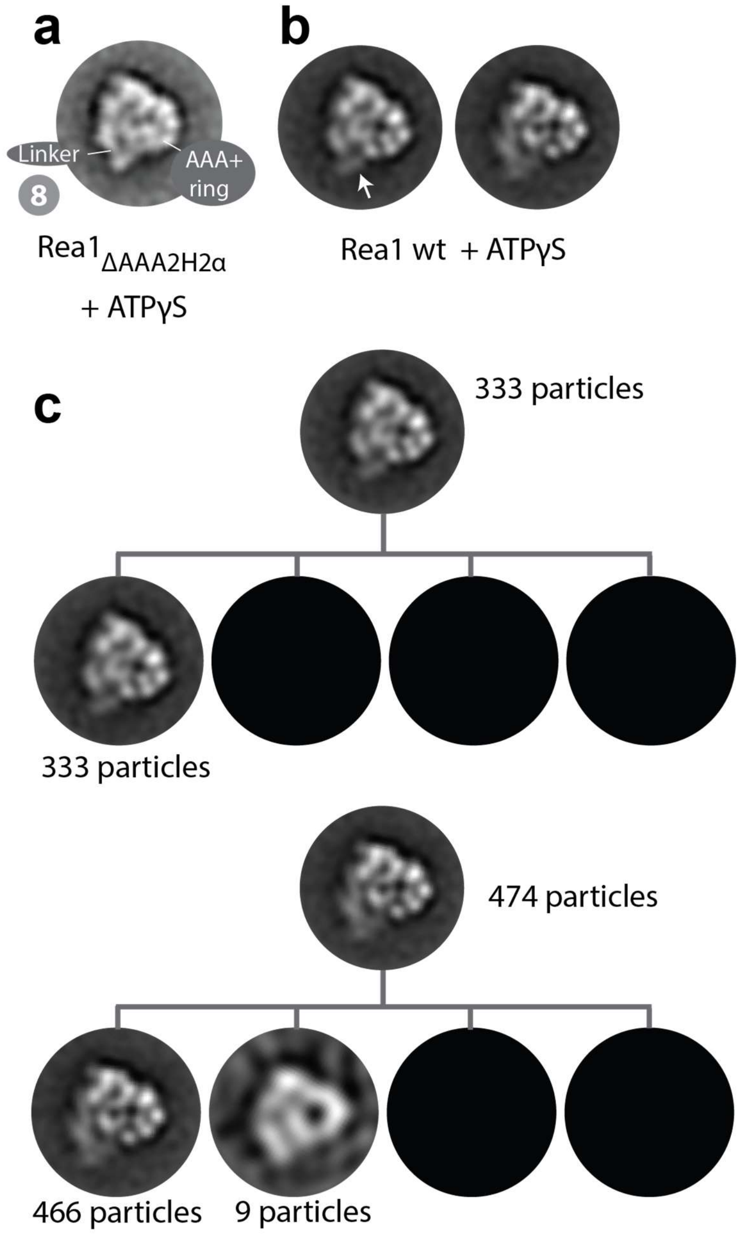

Supplementary Fig. 5: Linker remodelling state 8 is not stable in Rea1$_{wt}$. a State 8 as observed in Rea1$_{\Delta AAA2H2\alpha}$ in the presence of ATPγS. **b** Two 2D class averages obtained from a Rea1$_{wt}$ ATPγS data set. The two 2D classes are similar to state 8 of Rea1$_{\Delta AAA2H2\alpha}$, but thin stain for the linker tip (left, white arrow) or the complete linker (right) indicates increased structural flexibility. **c** The increased structural flexibility of the linker in b might be due to a mixture of well-folded state 8 particles and partially or completely unfolded particles. To rule out this possibility, we re-classified the particles in b into 4 sub-classes. The sub-classification brings back the original 2D classes confirming that the linker in these classes is too flexible to stably sample state 8.

a

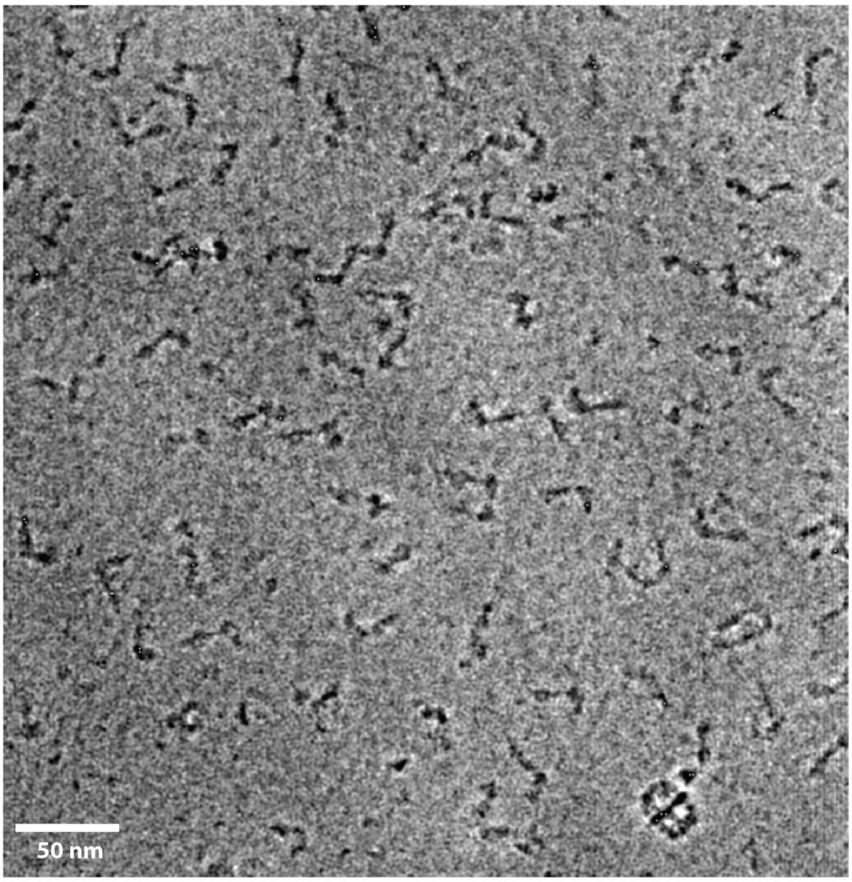

b

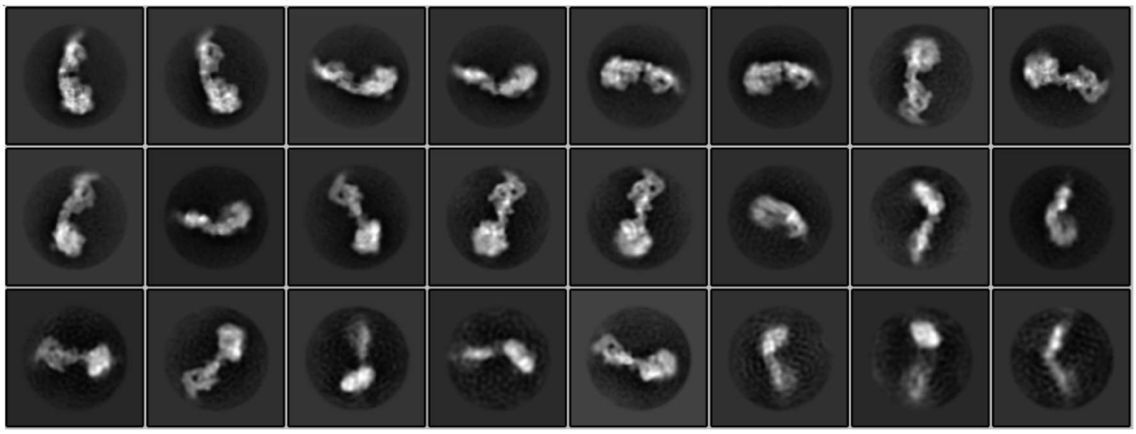

Supplementary Fig. 6: Rea1$_{\Delta AAA2H2\alpha}$ ATPγS cryoEM data. a Micrographs, **b** Representative 2D classes.

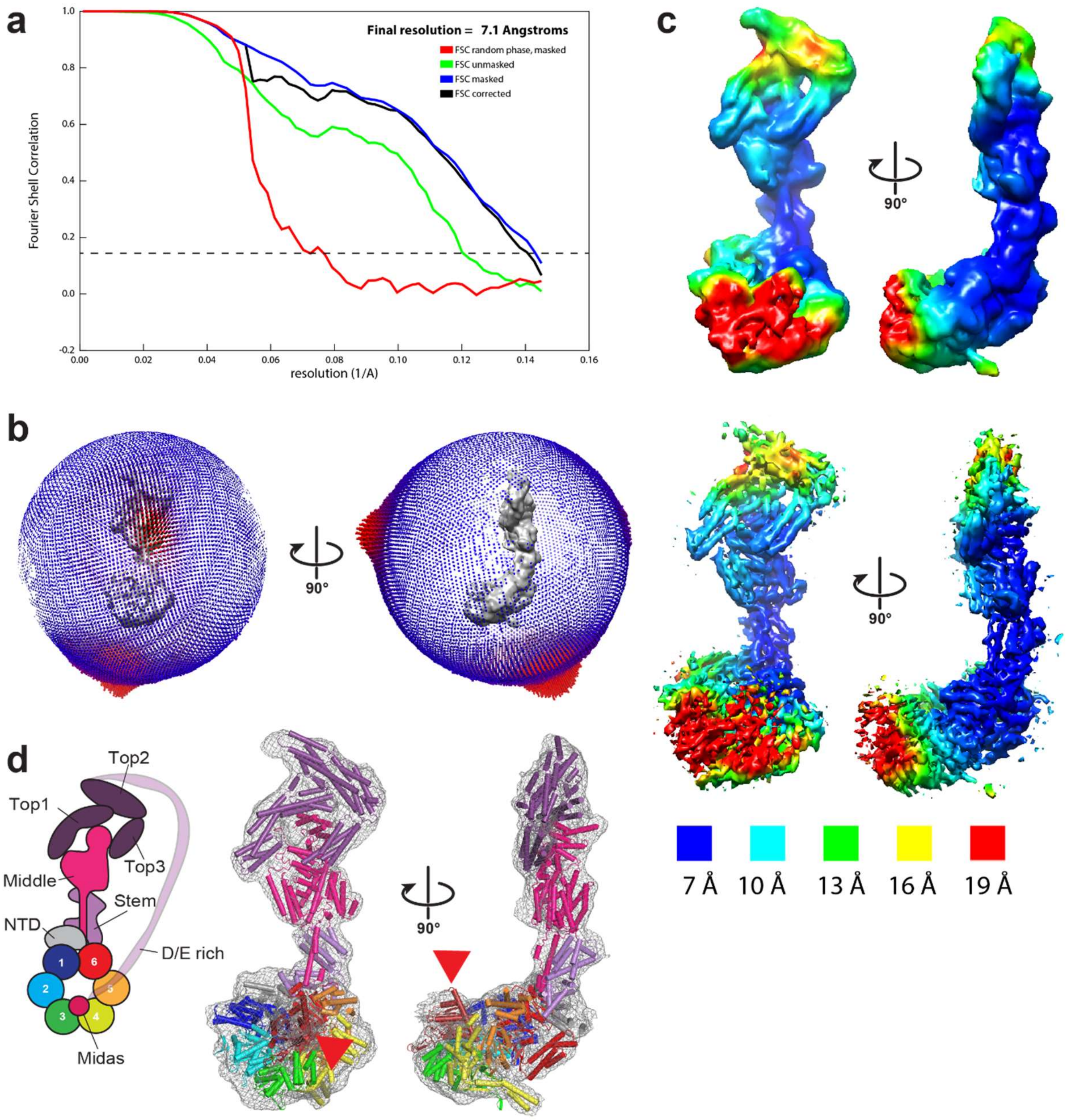

Supplementary Fig. 7: Overall quality of the Rea1$_{\Delta AAA2H2\alpha}$ ATPγS cryoEM map. a Fourier shell correlation (FSC) plot for half-maps of the 3D reconstruction. The 0.143 FSC criteria is indicated as horizontal dashed line. The final overall resolution is 7.1 Å. **b** Angular distribution of particles used in final reconstruction. **c** Local resolution map, upper panels: unsharpened map, lower panels: B-factor sharpened map. Secondary structure elements can be identified. **d** Schematic cartoon representation of structure (left) and match of structure in map (right). Red arrow heads highlight the AAA+ ring docked MIDAS domain. Cartoon adapted from Sosnowski et al. 2018[17].

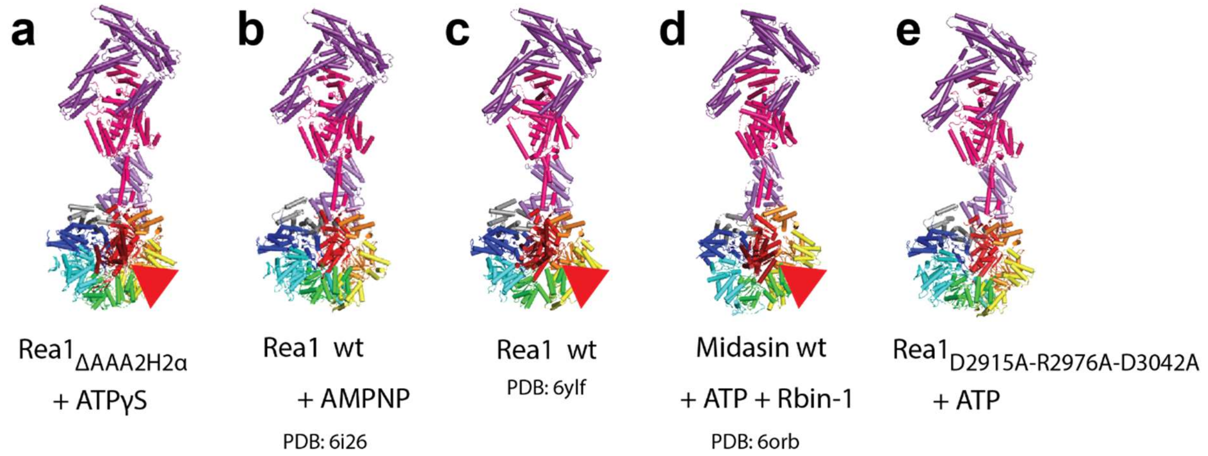

Supplementary Fig. 8: Comparison of the straight linker in the Rea1$_{ΔAAA2H2α}$ ATPγS cryoEM structure with other Rea1/Midasin cryoEM structures. **a** Straight linker in Rea1$_{ΔAAA2H2α}$ ATPγS structure (this study). **b** Rea1$_{wt}$ in the presence of AMPPNP (Sosnowski et al., 2018). **c** Rea1$_{wt}$ bound to a pre60S particle (Kater et al., 2020). **d** Midasin$_{wt}$ in the presence of ATP and the Rea1/Midasin inhibitor Rbin-1 (Chen at al., 2018). **e** Straight linker in Rea1$_{D2915A\text{-}R2976A\text{-}D3042A}$ (Conformation I) in the presence of ATP (this study). In all structures the linker adopts a similar straight conformation with respect to the AAA+ ring. Red arrow heads highlight the AAA+ docked MIDAS domain.

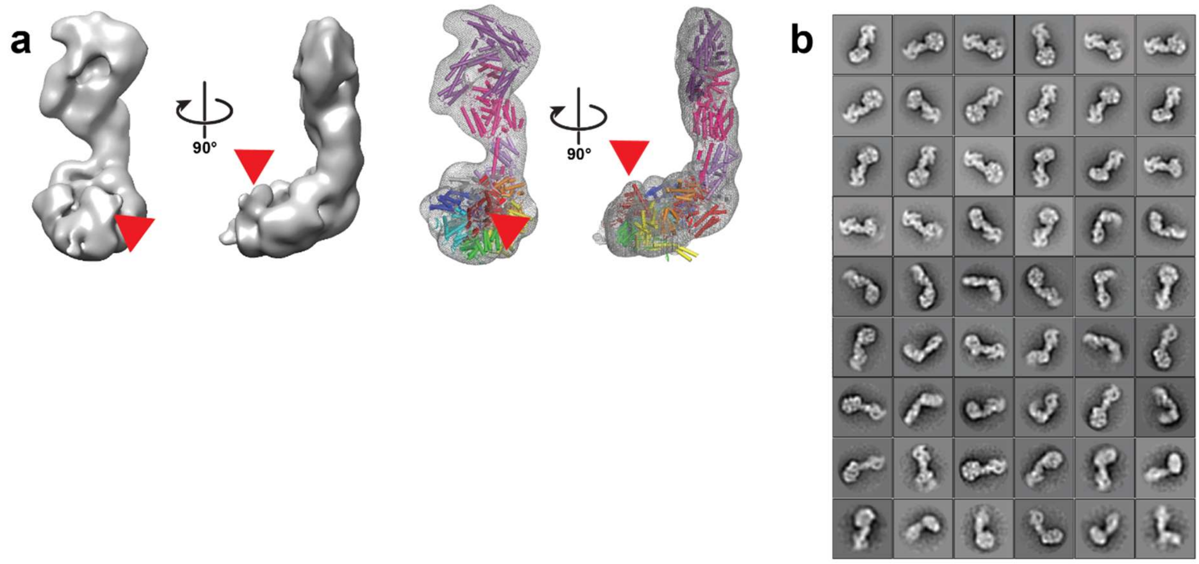

Supplementary Fig. 9: 3D reconstruction of the Rea1$_{\Delta AAA2H2\alpha}$ ATPγS negative stain EM data set. a Right panel: Final 3D reconstruction of the Rea1$_{\Delta AAA2H2\alpha}$ ATPγS negative stain EM data set. Left panel: Docking of the Rea1$_{\Delta AAA2H2\alpha}$ ATPγS cryoEM structure (Fig. 3a + Supplementary Fig. 7) into the negative stain EM map. The cryoEM structure, which represents the straight linker conformation, fits well into the negative stain EM map indicating that the negative stain data is not affected by staining artefacts. **b** 2D classification of the particles used in the final 3D reconstruction. The particles account for ≈45% of the total particles in the data set, which suggests that the straight linker conformation shown in a is the dominant structural state. AAA+ ring top views like in Figure 2 are absent indicating that they do not represent the straight linker conformation.

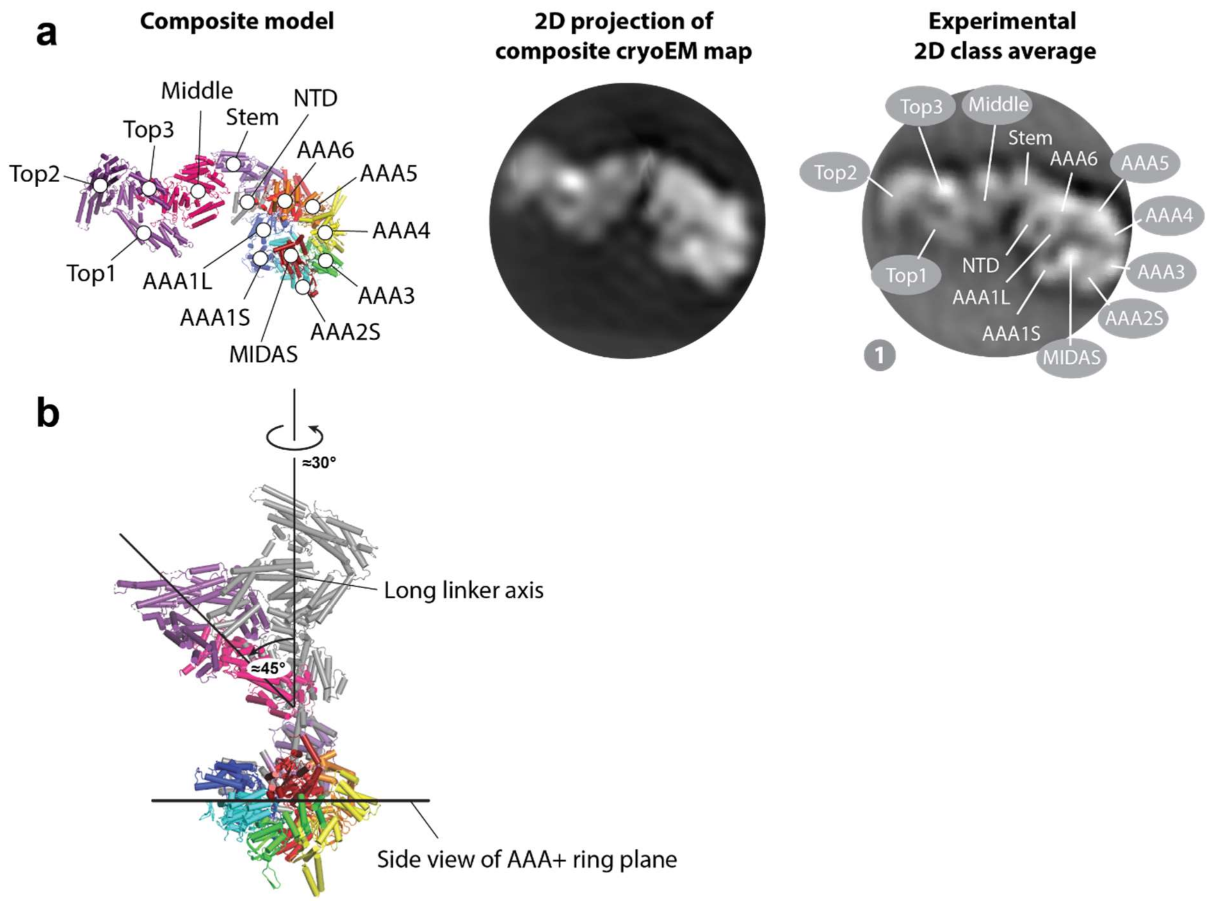

Supplementary Fig. 10: The linker in state 1 has moved with respect to the AAA+ ring compared to the straight linker conformation in the Rea1$_{\Delta AAA2H2\alpha}$ ATPγS cryoEM structure. a Combining the AAA+ ring map in the orientation used to assign the AAA+ sub-domains in state 1 with the linker map in the orientation used to assign linker sub-domains in state 1 by overlapping the linker stem area allowed us to create a structural composite model for state 1. **b** Aligning the AAA+ ring of this composite model with the AAA+ ring in the Rea1$_{\Delta AAA2H2\alpha}$ ATPγS cryoEM structure reveals that the linker top and middle domains have rotated by ≈30˚ and swung by ≈ 45˚ towards the AAA+ ring plane. Straight linker of the Rea1$_{\Delta AAA2H2\alpha}$ ATPγS cryoEM structure is shown in grey.

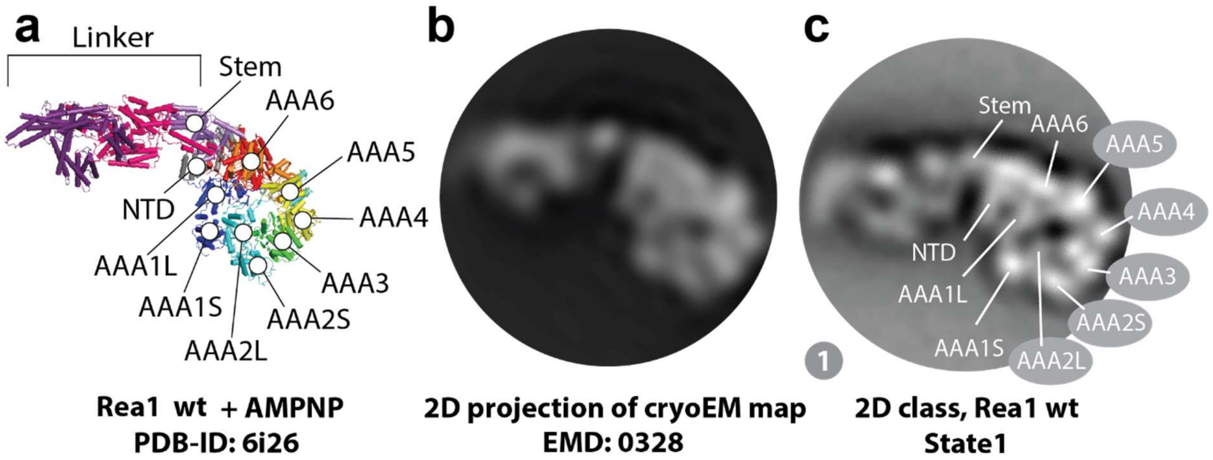

Supplementary Fig. 11: The AAA+ ring in the 2D class averages of Rea1$_{wt}$ does not harbour a docked MIDAS domain. a Structure of Rea1$_{wt}$ in the presence of AMPPNP (Sosnowski et al., 2018[17]). The AAA+ ring does not feature a docked MIDAS domain **b** 2D projection of the corresponding cryoEM map of a low pass filtered to 25 Å. **c** The AAA+ ring in the 2D class averages of Rea1$_{wt}$ (here state 1 as example) matches well with the projection in b suggesting the MIDAS domain is not docked onto the AAA+ ring.

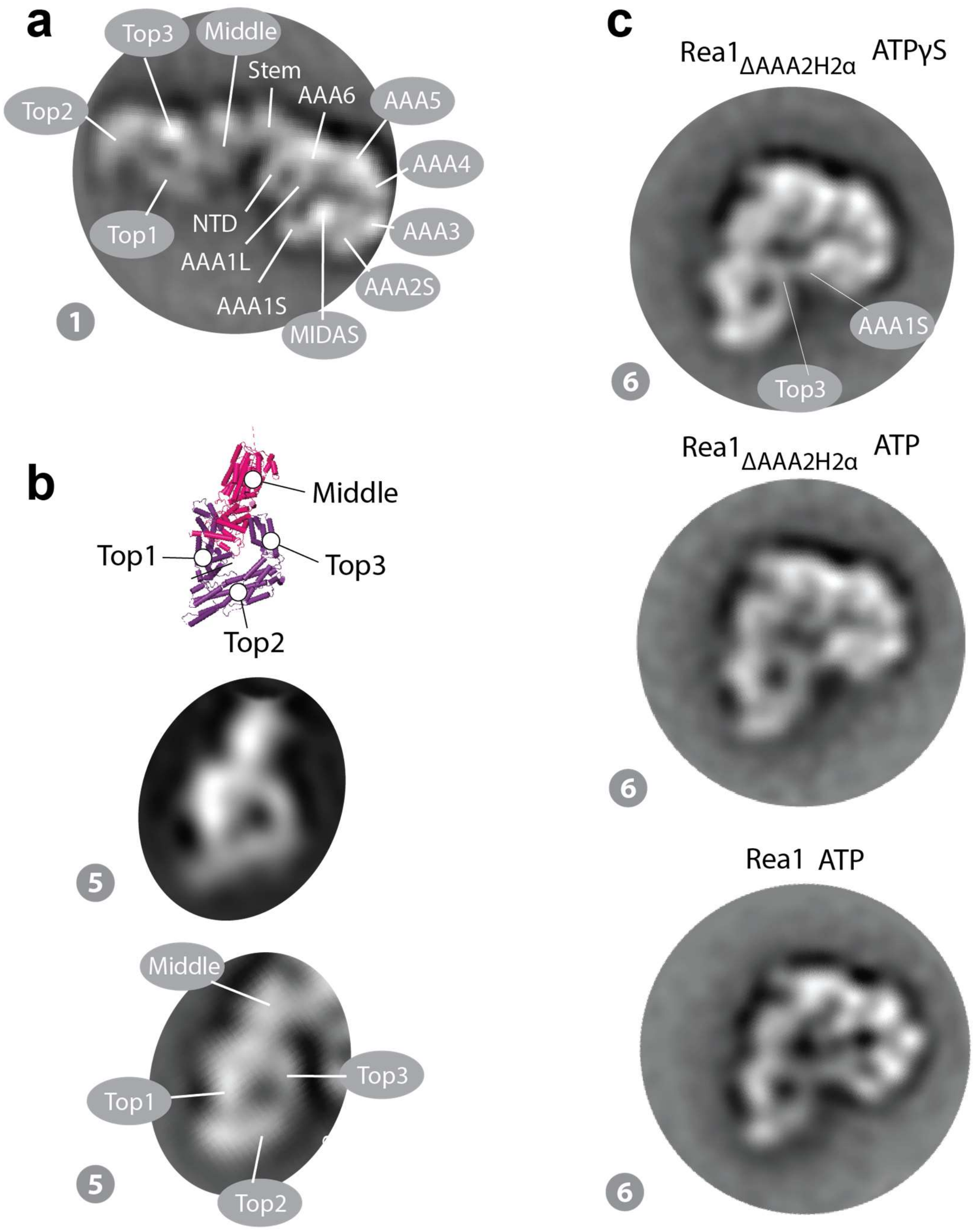

Supplementary Fig. 12: State 6 of the AAA+ ring engaged linker conformations features a connection between AAA1S and the linker top3 domain. a Domain assignments in the AAA+ ring and linker. **b** Two upper panels: Linker middle and top domains rotated into state 5 and corresponding cryoEM map projection (compare also Fig. 4c and d). Lower panel: Assignment of linker domains in state 5 based on the two upper panels. **c** The domain assignments in a and b suggest a connection between AAA1S and the linker top3 domain in state 6. The connection is visible in three independently collected data sets.

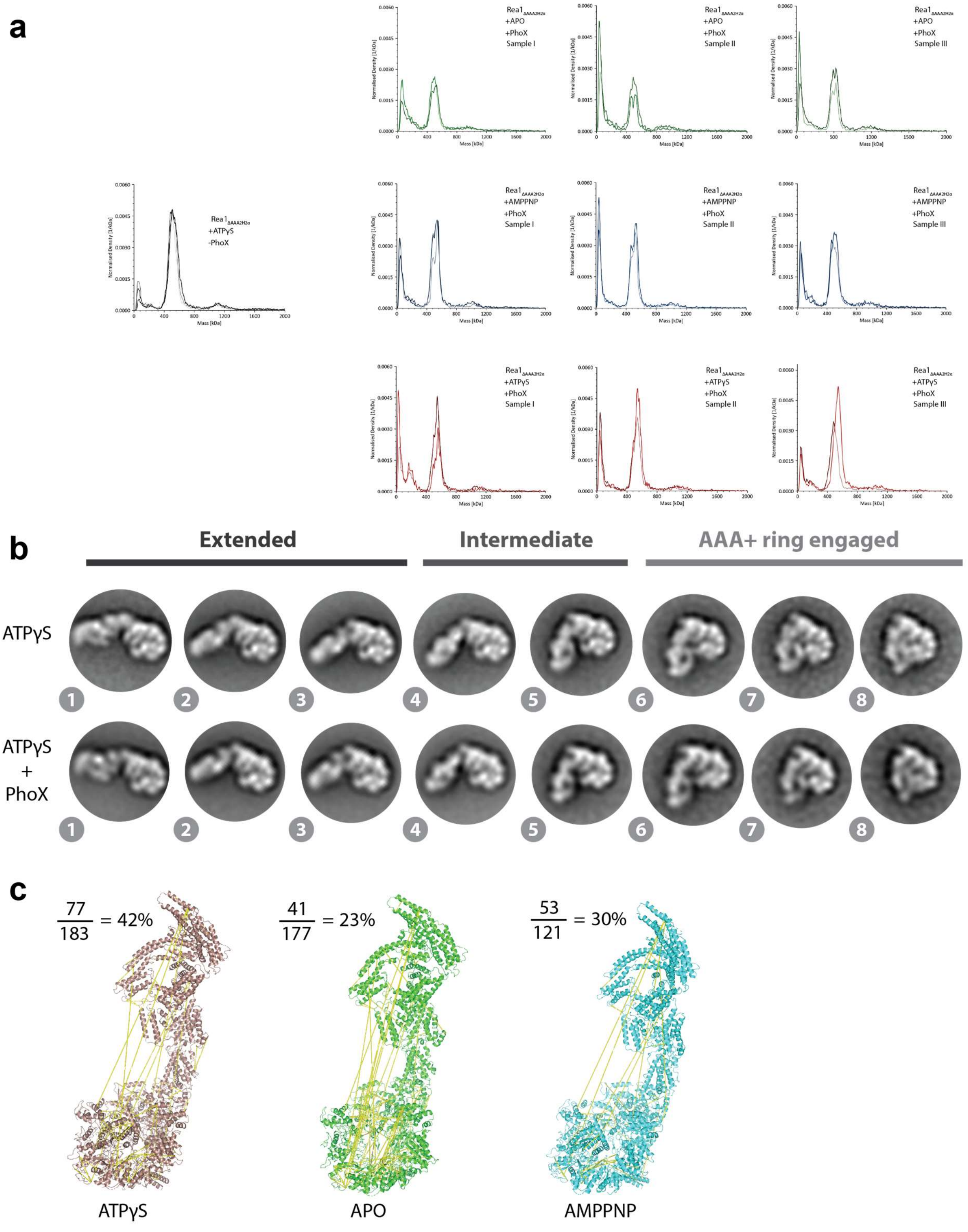

Supplementary Fig. 13: Validation of Rea1$_{\Delta AAA2H2\alpha}$ PhoX crosslinking. a Mass Photometry was used to analyse the amount of unspecifically crosslinked dimers. The theoretical mass for a Rea1$_{\Delta AAA2H2\alpha}$ monomer is 580 kDa. Solid lines represent the Gaussian-fits of major species. Compared to the non-crosslinked control (-PhoX) no significant stabilization or enrichment of Rea1$_{\Delta AAA2H2\alpha}$ dimers is observed in the three analysed PhoX crosslinked APO, AMPPNP and ATPγS replicates. This indicates that the detected crosslinks predominantly originate from intra-molecular interactions **b** A negative stain EM analysis demonstrates that presence of the PhoX crosslinker does not alter ATPγS induced linker

remodelling in Rea1$_{\Delta AAA2H2\alpha}$. **c** 182, 177 and 121 K-K unique crosslinks were detected in the PhoX crosslinked Rea1$_{\Delta AAA2H2\alpha}$ ATPγS, APO and AMPPNP samples. Out of these 77 (42%) (ATPγS), 41 (23%) (APO) and 53 (44%) (AMPPNP) can be assigned and distance-validated on the straight linker conformation as represented by the Rea1$_{\Delta AAA2H2\alpha}$ ATPγS cryoEM structure (compare Fig. 3a), which is expected to be the dominant structural state in all samples. A maximal Cα-Cα distance cut off of 25 Å was used to validate the crosslinks on the structure. The remaining crosslinks could be attributed to alternative Rea1 conformations, for which there is currently no 3D structural information available. The fact, that significant portions of the detected crosslinks can be assigned to a known structural state indicates a high quality of the crosslinking data.

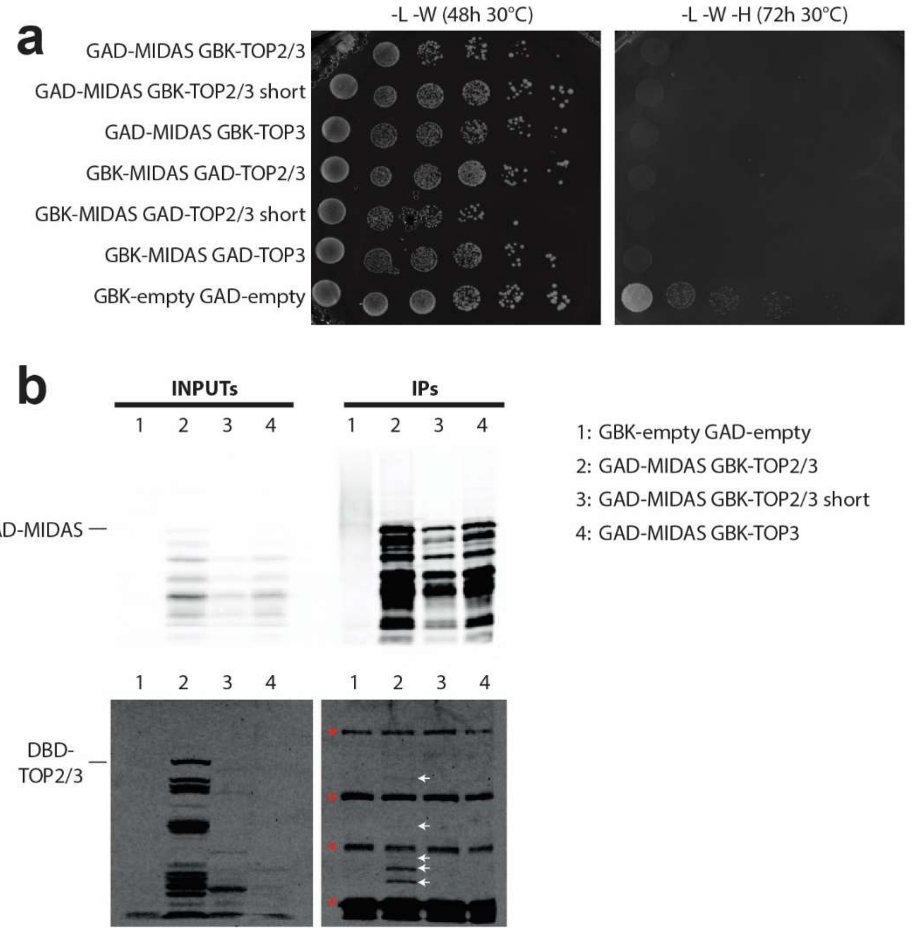

Supplementary Fig. 14: The MIDAS domain interacts with the linker top2/top3 region. a The MIDAS domain (E4623-S4910) was fused to 3xHA-GAL4AD (GAD, GAL4 activator domain) and the linker top2/top3 region (Y3557-N4041, short: I3601-N4041) or just the top3 region (D3786-E3905) to 3xMyc-GAL4BD (GBK, GAL4 DNA binding domain). Another set of plasmids reversed the GAD/GBK fusion constructs as indicated. The cells grew well on plates selecting the markers of the GAD/GBK plasmids (right panel, plates lacking leucine (L) and tryptophan (W)). Plating the cells on selective medium further lacking histidine (H) to check if the reporter *his3* expression has been induced, revealed that cells expressing the MIDAS/linker top2/top3 constructs grew slower than the empty vector control indicating toxicity to the cells. **b** In an alternative approach to probe for MIDAS-Linker top2/top3 interactions we carried out GAD-MIDAS immunoprecipitation experiments using anti-HA agarose beads. We checked for expression of the GAD-MIDAS and GBK-Linker top2/top3 constructs in the input lysates by anti-HA and anti-Myc western blot (left upper and left lower panel). The MIDAS and Linker top2/top3 constructs are prone to degradation. The degradation is especially pronounced in the case of the short linker top2/top3 as well as the linker top3 GBK constructs (lanes 3 and 4, left lower panel). Upper right panel: The GAD-MIDAS construct was pulled out from the input lysates via anti-HA agarose beads. The elution was subsequently analysed by anti-HA western blot. GAD-MIDAS can be detected in all elutions (lanes 2 – 4). Lower right panel: The eluates were also analysed by anti-myc western blot to probe for the presence of GBK-Linker top2/top3 constructs. In the case of the GBK-Linker top2/top3 constructs (lane 2), several degradation fragments can be detected (white arrows) indicating that parts of the Linker top2/top3 region are able to interact with the MIDAS domain. Signals marked with red asterisks likely result from detection of anti-HA antibody fragments present in the anti-HA agarose bead elutions by the secondary antibody.

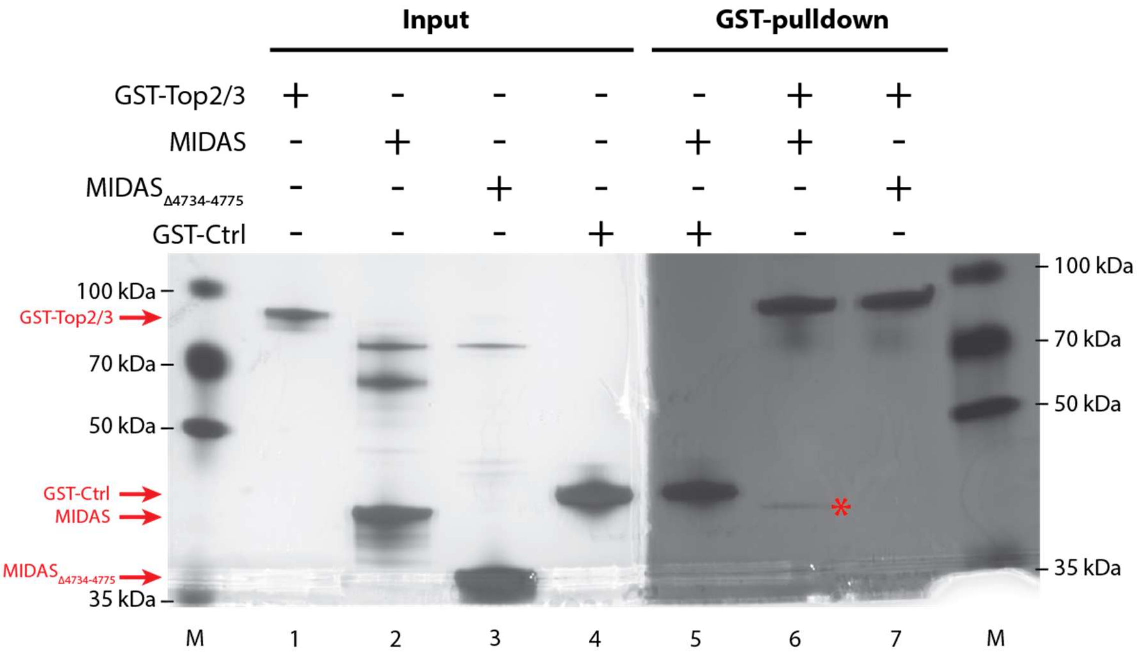

Supplementary Fig. 15: GST-linker top2/3 pulldown experiments with full length MIDAS and the MIDAS$_{\Delta4734\text{-}4775}$ construct. The samples were run on a SDS gel and silver stained. *C. thermophilum* GST-linker top2/3 pulls down the full length *C. thermophilum* MIDAS domain (lane 6, red asterisk), but not the MIDAS$_{\Delta4734\text{-}4775}$ construct (lane 7), which lacks the conserved T4733-K4778 loop (equivalent to the conserved *S. cerevisiae* MIDAS E4656-K4700 loop). The results suggest that the conserved loop region is involved in the linker top2/top3 – MIDAS interaction. M: marker, lanes 1, 2, 3 and 4: purified GST-linker top2/3 construct, MIDAS domain, MIDAS$_{\Delta4734\text{-}4775}$ and GST control. Lanes 5, 6 and 7: GST-control + MIDAS domain pulldown, GST-linker top2/3 + MIDAS domain pulldown and GST-linker top2/3 + MIDAS$_{\Delta4734\text{-}4775}$ pulldown. Samples were run together on the same SDS gel. Input and GST-pulldown lanes were separated and treated independently to avoid overdevelopment of the input lanes. The pulldown experiment was carried out once.

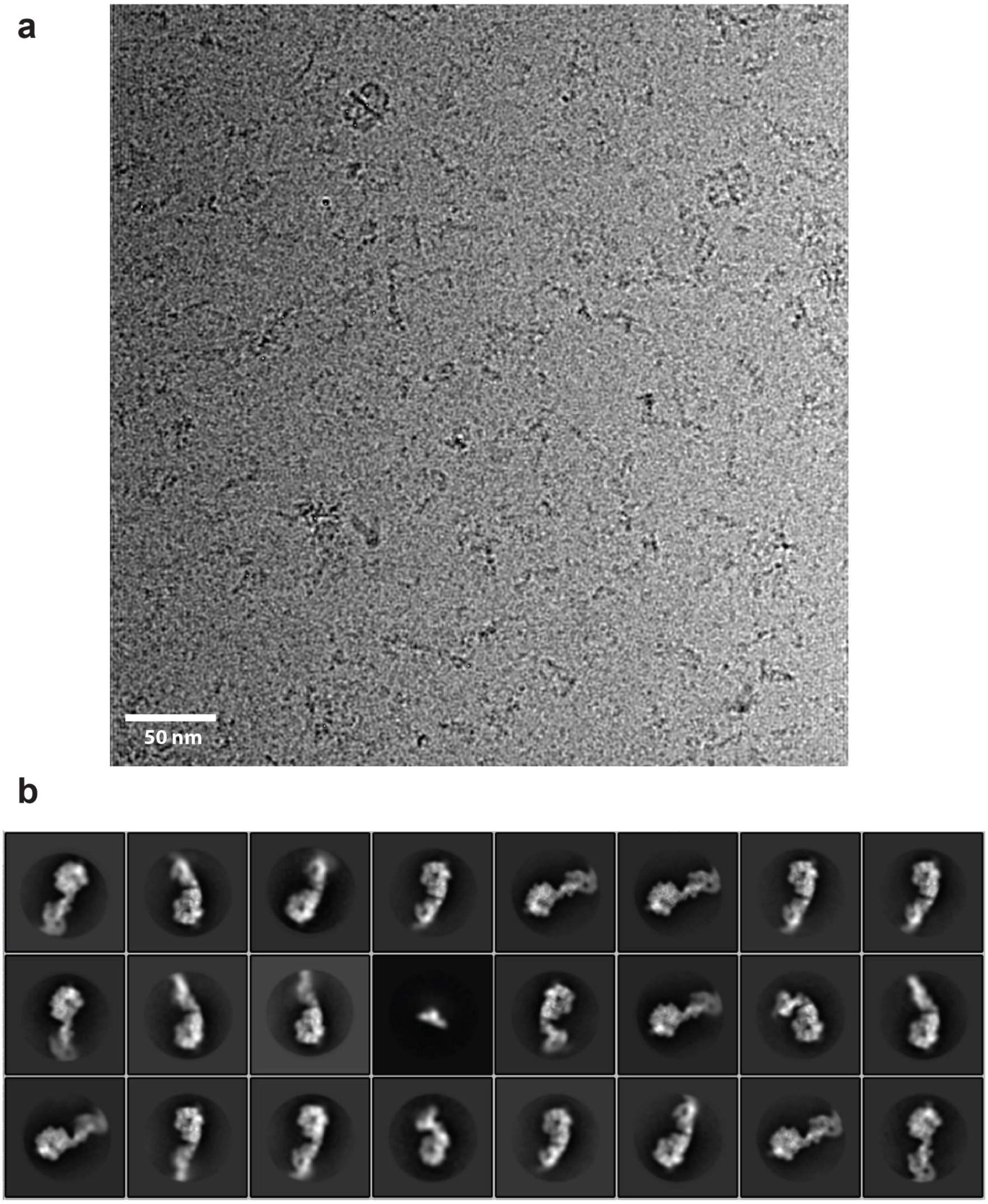

Supplementary Fig. 16: $Rea1_{D2915A\text{-}R2976A\text{-}D3042A}$ ATP cryoEM data. a Micrograph, **b** Representative 2D classes.

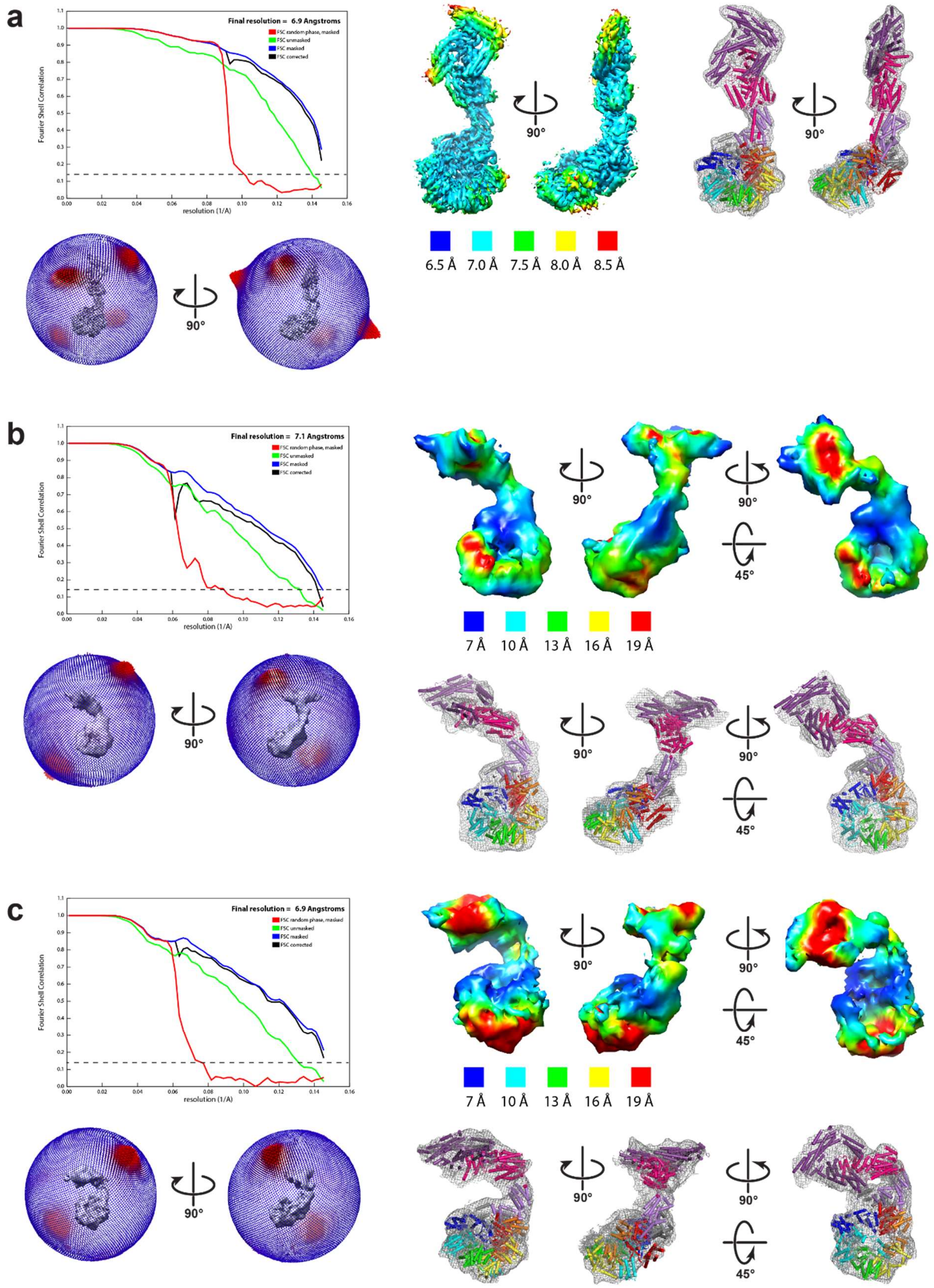

Supplementary Fig. 17: Overall quality of the Rea1$_{D2915A\text{-}R2976A\text{-}D3042A}$ ATP cryoEM maps. Fourier shell correlation (FSC) plot for half-maps of the 3D reconstructions (upper left panels, 0.143 FSC criteria is indicated as horizontal dashed line), angular distribution of particles used in final reconstruction (lower left panels), local resolution maps (middle and right panels) and match of structure in map (right and

lower right panels) of **a** Conformation I, **b** Conformation II and **c** Conformation III. In a the resolution is of sufficient quality to identify secondary structure elements. In b and c the resolution is of sufficient quality to dock in the linker middle-top domains and the linker stem-AAA+ring-NTD. Due to the use of binned data the 0.143 FSC criteria has not been reached in a and c. No substantial improvements in the resolution are to be expected with the unbinned data.

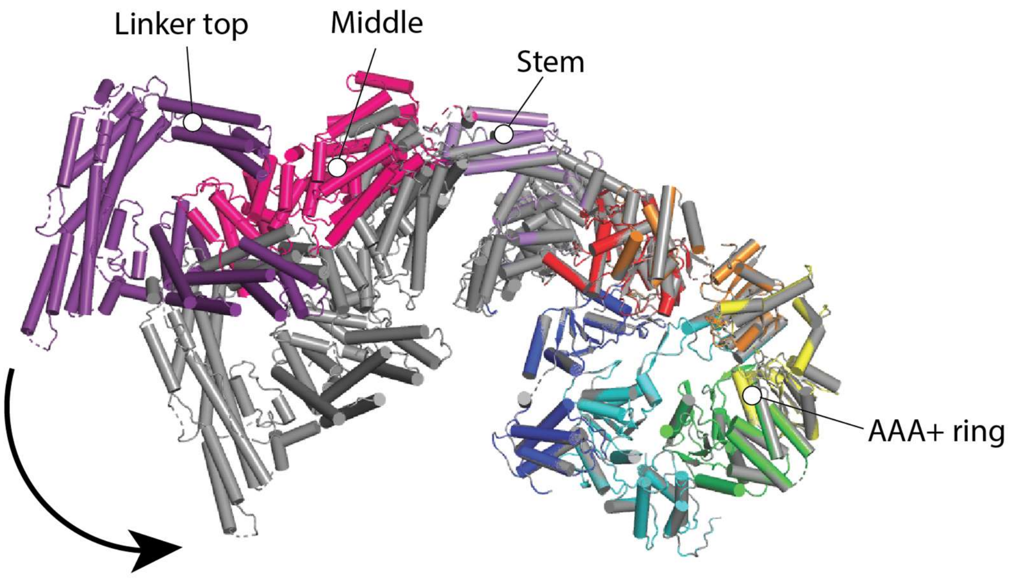

Supplementary Fig. 18: Conformations II and III of Rea1$_{D2915A\text{-}R2976A\text{-}D3042A}$ ATP are related by a swing of the linker middle and top domains towards the AAA+ ring. Conformation II is color coded, Conformation III is shown in grey. The structures have been aligned on the AAA+ rings. The black arrow indicates the swing towards the AAA+ ring. The region between the linker middle and stem domains acts as pivot point.

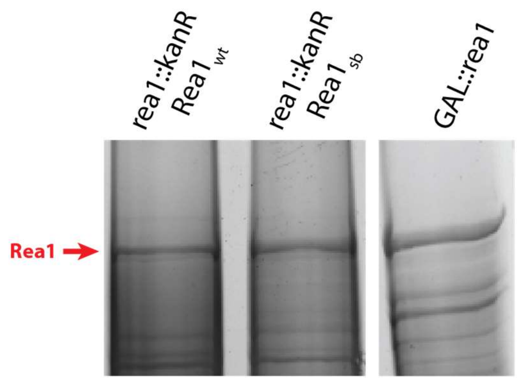

Supplementary Fig. 19: The Rea1$_{D2915A\text{-}R2976A\text{-}D3042A}$ mutant shows expression levels comparable to Rea1$_{wt}$. The lysates of *S. cerevisiae* strains harbouring centromeric plasmids expressing Rea1$_{wt}$ (rea1::kanR Rea1$_{wt}$) or the Rea1$_{D2915A\text{-}R2976A\text{-}D3042A}$ construct (rea1::kanR Rea1$_{sb}$) under the control of the endogenous Rea1 promotor and terminator regions were analysed by SDS-PAGE and silver staining. A *S. cerevisiae* strain overexpressing Rea1 in the presence of galactose (GAL::rea1) was processed in parallel to identify the band for Rea1 (red arrow). The expression levels of Rea1$_{wt}$ and Rea1$_{D2915A\text{-}R2976A\text{-}D3042A}$ are similar. The experiments were repeated four times.

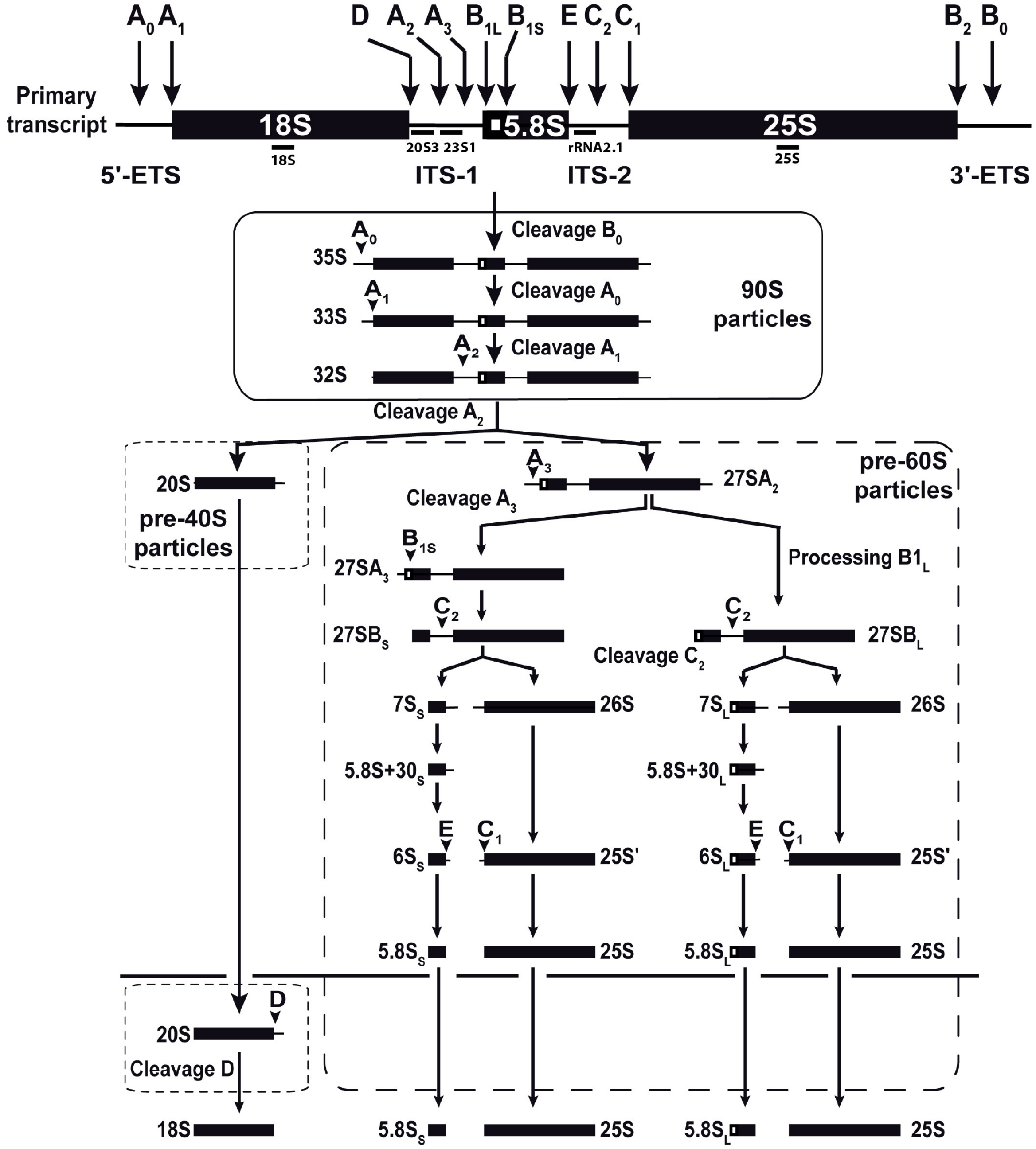

Supplementary Fig. 20: Cartoon of the pre-rRNA processing pathway in *S. cerevisiae*.

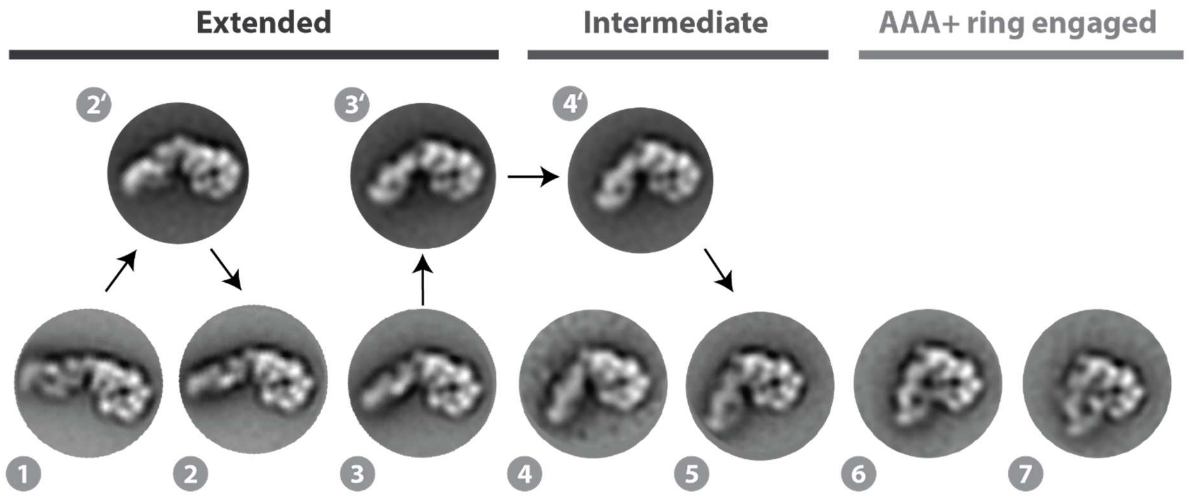

Supplementary Fig. 21: The swing and the rotation of the linker top and middle domains during linker remodelling are not strictly correlated. In addition to the linker states most commonly observed in our data sets (here states 1 – 7 of Rea1$_{wt}$ ATP as an example), additional extended and intermediate linker remodelling states were occasionally detected. Linker state 2′ represents a swing from state 1 towards the AAA+ ring without rotation. In state 3′ the linker middle and top domains are already fully rotated before reaching the proximity of the AAA+ ring. They swing without rotation via state 4′ to state 5.

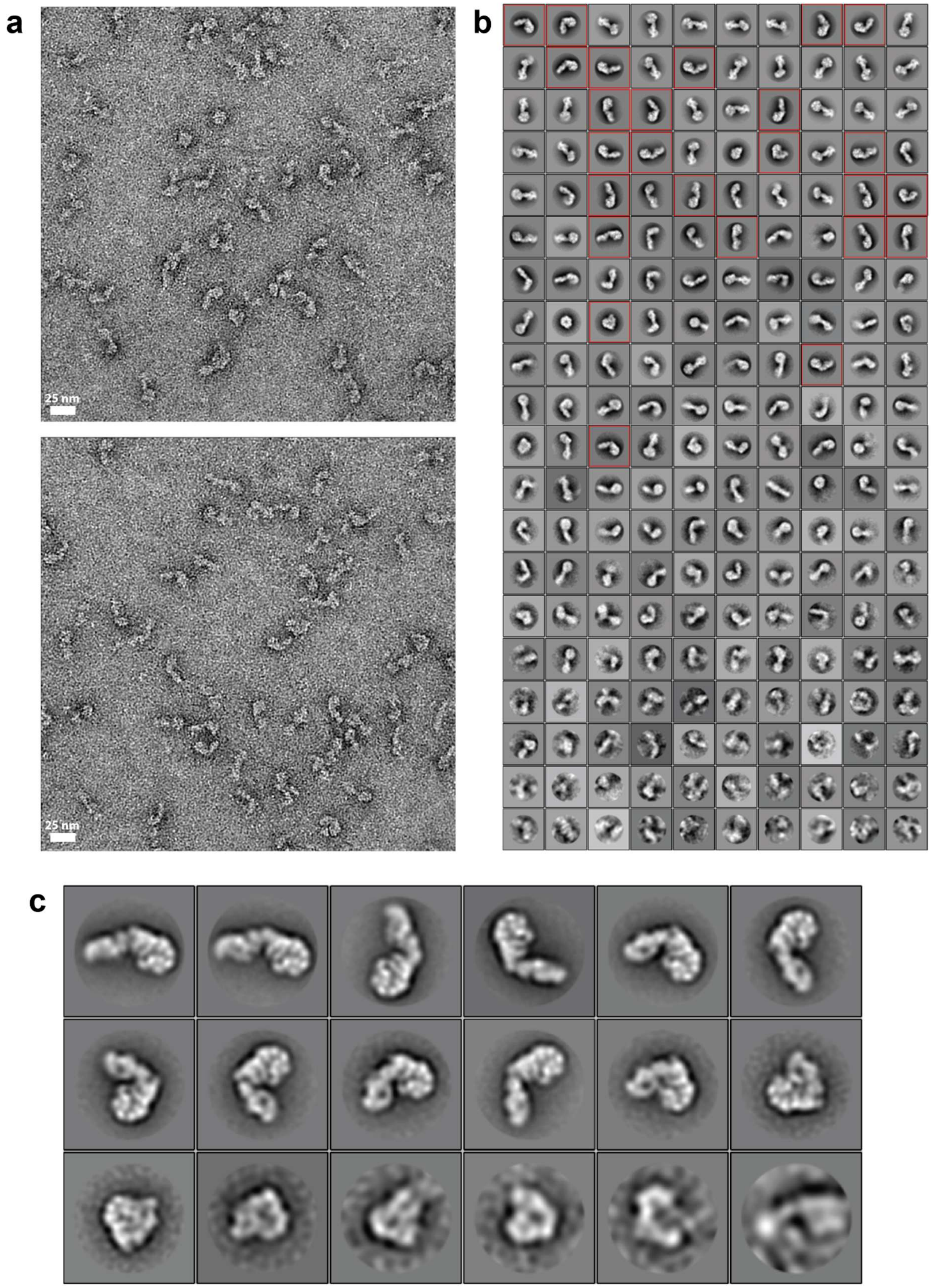

Supplementary Fig. 22: Quality of negative stain EM data. a Two representative micrographs of the Rea1$_{\Delta AAA2H2\alpha}$ ATPγS data set. **b** Initial 2D classification on 4x binned particles. 2D classes representing AAA+ ring top views of interest (red squares) were selected for a 2nd round of 2D classification. **c** 2nd round of 2D classification with the un-binned particles selected in b.

	#1 $Rea1_{\Delta AAA2H2\alpha}$ ATPγS (EMDB-50815)	#2 $Rea1_{sb}$ ATP Conformation I (EMDB-50816)	#2 $Rea1_{sb}$ ATP Conformation II (EMDB-50817)	#2 $Rea1_{sb}$ ATP Conformation III (EMDB-50818)
**Data collection and processing**				
Magnification	81000	81000	81000	81000
Voltage (kV)	300	300	300	300
Electron exposure (e–/Å^2)	45	45	45	45
Defocus range (μm)	1.8 – 3.4	1.8 – 3.4	1.8 – 3.4	1.8 – 3.4
Pixel size (Å)	0.862	0.862	0.862	0.862
Symmetry imposed	C1	C1	C1	C1
Initial particle images (no.)	230116	1035433	1035433	1035433
Final particle images (no.)	102746	322561	254169	242525
Map resolution (Å)	7.1	6.9	7.1	6.9
FSC threshold	0.143	0.143	0.143	0.143
Map resolution range (Å)	7-19	6.5 – 8.5	7-19	7-19

Supplementary Table 1: CryoEM data collection and validation statistics. $Rea1_{sb}$ = $Rea1_{D2915A\text{-}R2976A\text{-}D3042A}$

ATPγS

248 + 2318	1306 + 1873
249 + 1978	1311 + 1320
249 + 2318	1315 + 1320
293 + 304	1315 + 1548
293 + 436	1315 + 1548
304 + 2265	1318 + 3522
304 + 2318	1361 + 1439
304 + 321	1361 + 1548
304 + 361	1361 + 1873
304 + 377	1419 + 1463
304 + 436	1419 + 1548
304 + 441	1419 + 3104
321 + 377	1439 + 1548
316 + 436	1439 + 1978
316 + 441	1439 + 2179
316 + 492	1439 + 2186
316 + 499	1447 + 2186
321 + 377	1548 + 1593
361 + 859	1548 + 1600
361 + 1447	1548 + 1630
361 + 2186	1548 + 1873
361 + 436	1548 + 4662
377 + 859	1593 + 1601
377 + 2149	1767 + 2251
377 + 417	1924 + 1926
436 + 859	1924 + 1980
436 + 885	1980 + 2179
436 + 2265	1980 + 4570
436 + 489	1980 + 4576
436 + 492	1980 + 4785
436 + 499	1980 + 2186
436 + 505	1995 + 2458
441 + 2265	2149 + 2186
441 + 2318	2316 + 2946
441 + 505	2318 + 2939
489 + 3669	2318 + 2946
492 + 505	2318 + 4452
499 + 885	2416 + 2424
499 + 4792	2416 + 2431
505 + 885	2416 + 2452
505 + 956	2421 + 2452
505 + 4792	2424 + 2452
505 + 611	2584 + 2855
611 + 859	2584 + 2864
611 + 4662	2677 + 4469
611 + 619	2701 + 3307
611 + 668	2701 + 3310
613 + 619	2705 + 3310
613 + 673	2707 + 2793
618 + 668	2707 + 3195
619 + 668	2793 + 3195
619 + 673	2793 + 3334
668 + 673	2801 + 3334
619 + 2149	2939 + 2946
818 + 859	2939 + 2958
859 + 885	3104 + 3245
859 + 1980	3161 + 3180
859 + 2149	3161 + 3195
859 + 4662	3166 + 3180
859 + 4668	3180 + 3396
859 + 4785	3195 + 3334
866 + 885	3220 + 3455
866 + 4662	3220 + 3459
885 + 956	3303 + 3310
885 + 3104	3356 + 3522
941 + 956	3455 + 3486
1005 + 1017	3514 + 3527
1005 + 1020	3522 + 3527
1005 + 1038	3569 + 4662
1005 + 1063	3586 + 4163
1010 + 1020	3586 + 4169
1020 + 2318	3586 + 4171
1020 + 3669	3586 + 4180
1089 + 1265	3657 + 3662
1103 + 1439	3669 + 3677
1103 + 3104	3793 + 3907
1244 + 1306	3954 + 3961
1244 + 1311	3955 + 4662/4668
1244 + 1318	4163 + 4171
1244 + 1361	4459 + 4469
1244 + 1419	4635 + 4719
1244 + 1548	4635 + 4792
1265 + 1306	4662 + 4664
1265 + 1315	4662 + 4785
1265 + 1439	4662 + 4792
1265 + 1548	4719 + 4753
1265 + 1873	4753 + 4785
1265 + 4662	4753 + 4792
1306 + 1315	4780 + 4785
1306 + 1361	4784 + 4792
1306 + 1548	4719 + 4792

APO

83 + 2931	1094 + 1548
147 + 3669	1103 + 1419
203 + 1422	1103 + 3104
248 + 2318	1243 + 2458
249 + 2318	1244 + 1306
249 + 304	1244 + 1315
249 + 316	1244 + 1419
249 + 321	1244 + 1548
249 + 436	1244 + 1873
293 + 859	1265 + 1306
293 + 377	1265 + 1315
304 + 859	1265 + 1320
304 + 866	1265 + 1361
304 + 885	1265 + 1419
304 + 1265	1265 + 1548
304 + 2265	1265 + 1873
304 + 2318	1265 + 4662
304 + 2946	1306 + 1315
304 + 4662	1306 + 1345
304 + 321	1306 + 1548
304 + 361	1306 + 1873
304 + 377	1310 + 1320
304 + 436	1310 + 4671
304 + 441	1311 + 1320
316 + 2265	1315 + 1320
316 + 377	1315 + 1548
316 + 436	1345 + 1873
316 + 441	1361 + 1548
316 + 492	1419 + 1447
321 + 859	1419 + 1463
321 + 885	1419 + 1548
321 + 2265	1439 + 1548
321 + 377	1447 + 1548
321 + 417	1451 + 1548
321 + 436	1463 + 1548
361 + 859	1548 + 1593
361 + 2186	1548 + 1600
361 + 377	1548 + 1630
361 + 436	1548 + 1873
377 + 859	1548 + 1980
377 + 866	1924 + 1926
377 + 885	1995 + 2458
377 + 2149	2149 + 2186
377 + 2265	2316 + 2318
377 + 2318	2316 + 2946
377 + 4662	2316 + 4452
377 + 417	2318 + 2939
377 + 436	2318 + 2946
377 + 441	2416 + 2424
377 + 611	2424 + 2452
436 + 859	2458 + 2958
436 + 885	2677 + 4469
436 + 2265	2701 + 3310
436 + 489	2793 + 3195
436 + 499	2793 + 3334
436 + 505	2939 + 2946
436 + 668	3104 + 3245
441 + 2265	3161 + 3180
441 + 2318	3161 + 3195
489 + 3669	3166 + 3180
505 + 885	3180 + 3396
505 + 956	3195 + 3334
611 + 859	3356 + 3522
611 + 1265	3455 + 3486
611 + 668	3514 + 3527
613 + 619	3522 + 3527
613 + 668	3586 + 4163
613 + 673	3586 + 4169
618 + 668	3586 + 4171
668 + 4785	3586 + 4180
668 + 673	3657 + 3662
818 + 859	3669 + 3677
859 + 1265	4163 + 4171
859 + 1980	4169 + 4180
859 + 2149	4359 + 4370
859 + 2265	4459 + 4469
859 + 4662	4545 + 4570
859 + 4785	4570 + 4662
866 + 885	4635 + 4792
866 + 4662	4662 + 4753
885 + 956	4662 + 4785
885 + 3104	4662 + 4792
1005 + 1017	4719 + 4792
1005 + 1020	4753 + 4785
1005 + 1265	4753 + 4792
1005 + 1548	4780 + 4785
1020 + 2318	
1020 + 2707	
1020 + 3669	
1089 + 1103	
1094 + 1265	

AMPPNP

147 + 3669	2677 + 4469
203 + 1422	2701 + 3307
248 + 2318	2701 + 3310
249 + 1980	2793 + 3195
249 + 2318	2793 + 3334
293 + 436	2939 + 2946
304 + 2265	3104 + 3245
304 + 2318	3161 + 3180
304 + 321	3161 + 3195
304 + 377	3166 + 3180
304 + 436	3180 + 3396
304 + 441	3195 + 3334
316 + 436	3356 + 3522
316 + 441	3455 + 3486
316 + 492	3522 + 3527
321 + 866	3586 + 4163
321 + 377	3586 + 4169
361 + 859	3586 + 4171
361 + 2186	3586 + 4180
377 + 859	3657 + 3662
377 + 2149	3669 + 3677
436 + 885	4163 + 4171
436 + 2265	4359 + 4370
436 + 489	4459 + 4469
436 + 499	4545 + 4570
436 + 505	4635 + 4719
441 + 2265	4662 + 4785
489 + 3669	4753 + 4785
499 + 4792	4753 + 4792
505 + 885	4780 + 4785
505 + 956	
505 + 4792	
611 + 859	
611 + 619	
611 + 668	
613 + 619	
613 + 668	
613 + 673	
618 + 668	
668 + 673	
818 + 859	
859 + 2149	
859 + 4785	
885 + 956	
885 + 3104	
1005 + 1017	
1005 + 1020	
1017 + 3669	
1020 + 2316	
1103 + 3104	
1244 + 1306	
1244 + 1311	
1244 + 1315	
1244 + 1318	
1244 + 1548	
1265 + 1306	
1265 + 1315	
1265 + 1548	
1265 + 1873	
1306 + 1315	
1306 + 1548	
1306 + 1873	
1311 + 1320	
1311 + 4671	
1315 + 1320	
1315 + 1548	
1419 + 1463	
1419 + 1548	
1439 + 1548	
1439 + 2186	
1447 + 2186	
1548 + 1600	
1548 + 1630	
1548 + 1873	
1548 + 4662	
1767 + 2251	
1924 + 1926	
1980 + 4570	
1995 + 2458	
2149 + 2186	
2316 + 2946	
2316 + 4452	
2318 + 2939	
2318 + 2946	
2416 + 2424	
2416 + 2431	
2416 + 3455	
2421 + 2452	
2424 + 2452	
2458 + 2958	
2622 + 2839	

Supplementary Table 2: List of validated K-K crosslinks in the Rea1$_{\Delta AAA2H2\alpha}$ ATPγS, APO and AMPPNP mass spectrometry data sets. Crosslinks between the linker top2 region and the MIDAS domain are highlighted in yellow. K-K crosslinks are labelled according to *S. cerevisiae* Rea1$_{wt}$ numbering. Please note that in the data sets submitted to the ProteomeXchange Consortium Rea1$_{\Delta AAA2H2\alpha}$ numbering will be used.

GK1	AGGTGTGTCTAGACTTAGTTCCGAAAAGTGCCACCTGGGTCCTTTTC
GK2	AGAAAGATATAAAATTTTGCGGAAATGTGCGCGGAACCCCTATTTG
GK3	GCAAAATTTTATATCTTTCTTGGAATC
GK4	CACGATATACTTACATTCTCAGCAGATTATAACGGTTTTA
GK5	CTGCTGAGAATGTAAGTATATCGTGAAAACCAAGAAGAG
GK6	AACTAAGTCTAGACACACCTCCTGGTG
GK7	TGGCATCCAGCTAAGTATATCGTGAAAACCAAG
GK8	CTATCCTGGGACATTCTCAGCAGATTATAAC
GK9	ATCTGCTGAGAATGTCCCAGGATAGAATTTTGTTAG
GK10	TCACGATATACTTAGCTGGATGCCAGGTCTGTAAAG
GK11	GCCGGATCATAGGGAGAATCGCCTCCCGACAACTGTAAAACATCGGTAAATTC
GK12	CTCAATCCCCTAGGGGTTCTGGGTTCTGTTCTCCGCAGCCACCATCTG
GK13	TTTTACAGTTGTCGGGAGGCGATTCTCCCTATGATCCGGCAATACAC
GK14	GGCTGCGGAGAACAGAACCCAGAACCCCTAGGGGATTGAGGTGCGTCGTCATC
GK15	CAGATTCTCCCTATGCACCGGCAATACAC
GK16	GTGTATTGCCGGTGCATAGGGAGAATCTG
GK17	CTAGGGTATATGCAACAGGCATGTCTATTG
GK18	CAATAGACATGCCTGTTGCATATACCCTAG
GK19	GGTTATTCAAAATTTGCTGCATTAAATGATATCCTC
GK20	GAGGATATCATTTAATGCAGCAAATTTTGAATAACC
GK21	GGAGCCCACATCGTGATGGTGGACGCCTACAAGCCGACGAAGTAAGGTACCGAGCTCGGATCCAC
GK22	CTTCGTCGGCTTGTAGGCGTCCACCATCACGATGTGGGCTCCTGAATTATCACTTGCCTTATCATC
GK23	GGAGCCCACATCGTGATGGTGGACGCCTACAAGCCGACGAAGGGTACTGATGATAACTGGTTATCAGCTAG
GK24	ACCCTTCGTCGGCTTGTAGGCGTCCACCATCACGATGTGGGCTCCTTCGGATATAGATTCAATTCCATC
GK25	GCTTTTAGAGGGAATGTCTAAAGGTGAAGAATTATTC
GK26	GTCACCACTGCCAGATCCACCATGGGTAATACCAGCAGCAG
GK27	CATGGTGGATCTGGCAGTGGTGACTTGAAAGATCTTGCTAATC
GK28	TAGACATTCCCTCTAAAAGCCTTGAGGATAC
GK29	CTATTGCTCGATCAAGTGAATTTAGCAAC
GK30	GTTGCTAAATTCACTTGATCGAGCAATAG
GK31	GTTTTGGATCAATTAAATCTTGCCCCAAC
GK32	GTTGGGGCAAGATTTAATTGATCCAAAAC
GK33	CTACTTGATCAAATATCGCTAGCAGATGATTC
GK34	GAATCATCTGCTAGCGATATTTGATCAAGTAG
GK35	GTGGGTTTTATTAGATCAAATGAATTTAGCCTC
GK36	GAGGCTAAATTCATTTGATCTAATAAAACCCAC
GK37	CTATTGCTCGATCAAGTGAATTTAGCAAC
GK38	AGATCTGAACGTTGATACCTTATCAGGTTTATCAAG
GK39	GCTCGAGACCtccAATATGAGCGTCACCTTTAGTTG
GK40	TCATATTggaGGTCTCGAGCACCACCAC
GK41	AGGTATCAACGTTCAGATCTTCCTCGC
TK1	AGGGCGGATCCCACATCTCTGATGAACAATTG
TK2	CTTCGGACCGTTAGCTGTTCACCTCTGCGAAC
TK3	GAACAGCTAACGGTCCGAAGCGCGCGGAATTC
TK4	CAGAGATGTGGGATCCGCCCTGAAAATAAAGATTC
TK5	TTTCAGGGCGGATCCGGTGATCAGAAAGCCATAAGTG
TK6	GCGCGCTTCGGACCGACCTTGAGAGGCTATTTGGAC
TK7	ATAGCCTCTCAAGGTCGGTCCGAAGCGCGCGGAATTC
TK8	GGCTTTCTGATCACCGGATCCGCCCTGAAAATAAAGATTC
DV1	TGACAGGCTCGGGCAGCGGTAGTAAACGTGCTTACCAG

DV2	CAATTGAGGTTGATATTGACAGGCTCGGGCAGCGGTAGTAAACG
OHA529	CCTCTGAGTTAATTTGACATCGAATTCTACTCTTATTATTTTAAGGTACCGAATTCGAGCTCGTTTAAAC
OHA530	ATTAACCTTTGGTTAACTACGTCTAAATCTAACAAAATTCTATCCTGGGACATTTTGAGATCCGGGTTTT
OHA532	GGCATTGATTTTAAATGCACTTGAACCG
OHA533	ACATGACCAGTGATGGAACGGC
OHA534	GTCCGTTCTCTTAACAACCAAAGCC
OHA543	GTCAAGACAAAAAGATTCAAATAAATAAAGGAGACAACATTTTCAAAAACCGGATCCCCGGGTTAATTAA
OHA544	TTCAAGTATATCAGTACATTATTCTACATAAAAACAACAACAGTTTTTCGAATTCGAGCTCGTTTAAAC
OHA545	TTTCCTCTCTGGACACTTGTCC
OHA546	CCGCCAGAATGGTTTGAAAAGG
OHA553	GATCAGAAGGTGGAGGAATCAGGGGTTTTAGAGCTAG
OHA554	CTAGCTCTAAAACCCCTGATTCCTCCACCTTCT
OHA551	TTTTTATATTTGTAACCTCGATAAACACACACTAACACAATATACAGATGCGGATCCCCGGGTTAATTAAC
OHA552	AGTTGAGCCTCTTTCTTTTGTTTCTTAGAAGGTGGAGGAATCAGGGTGGAGCACTGAGCAGCGTAATCTGG
OHA559	AGCTCATAGAGTGGTCAACGTC
OHA560	GAAGGTATATGGCACTGGATCG
OHA631	CAGATTACGCTCATATGGAAAGATCGTTAGAAGAATCCCGTG
OHA632	CGAGCTCGATGGATCCTTAGCTGGATGCCAGGTCTG
OHA633	CAGATTACGCTCATATGTATTCCAACGAAAATAAACTAAAAC
OHA634	CGAGCTCGATGGATCCTTAATTTTTGGCTAAGCTATGC
OHA635	CAGATTACGCTCATATGATTGACACGGTAGCAAGTAACA
OHA636	CGAGCTCGATGGATCCTTAATTTTTGGCTAAGCTATGCAAT
OHA637	CAGATTACGCTCATATGGATATTGTTCATTCTTCATTAAAAG
OHA638	CGAGCTCGATGGATCCTTATTCCTTTTTAATAGGACCCC
OHA639	AGGAGGACCTGCATATGGAAAGATCGTTAGAAGAATCCCGTG
OHA640	GCAGGTCGACGGATCCTTAGCTGGATGCCAGGTCTG
OHA641	AGGAGGACCTGCATATGTATTCCAACGAAAATAAACTAAAAC
OHA642	GCAGGTCGACGGATCCTTAATTTTTGGCTAAGCTATGC
OHA643	AGGAGGACCTGCATATGATTGACACGGTAGCAAGTAACA
OHA644	GCAGGTCGACGGATCCTTAATTTTTGGCTAAGCTATGCAAT
OHA645	AGGAGGACCTGCATATGGATATTGTTCATTCTTCATTAAAAG
OHA646	GCAGGTCGACGGATCCTTATTCCTTTTTAATAGGACCCC
18S	CATGGCTTAATCTTTGAGAC
20S.3	TTAAGCGCAGGCCCGGCTGG
23S.1	GATTGCTCGAATGCCCAAAG
rRNA2.1	GGCCAGCAATTTCAAGTTA
25S	CTCACGACGGTCTAAACCC

Supplementary Table 3: Table of oligonucleotide sequences used in the study.

Source Data for Supplementary Figures

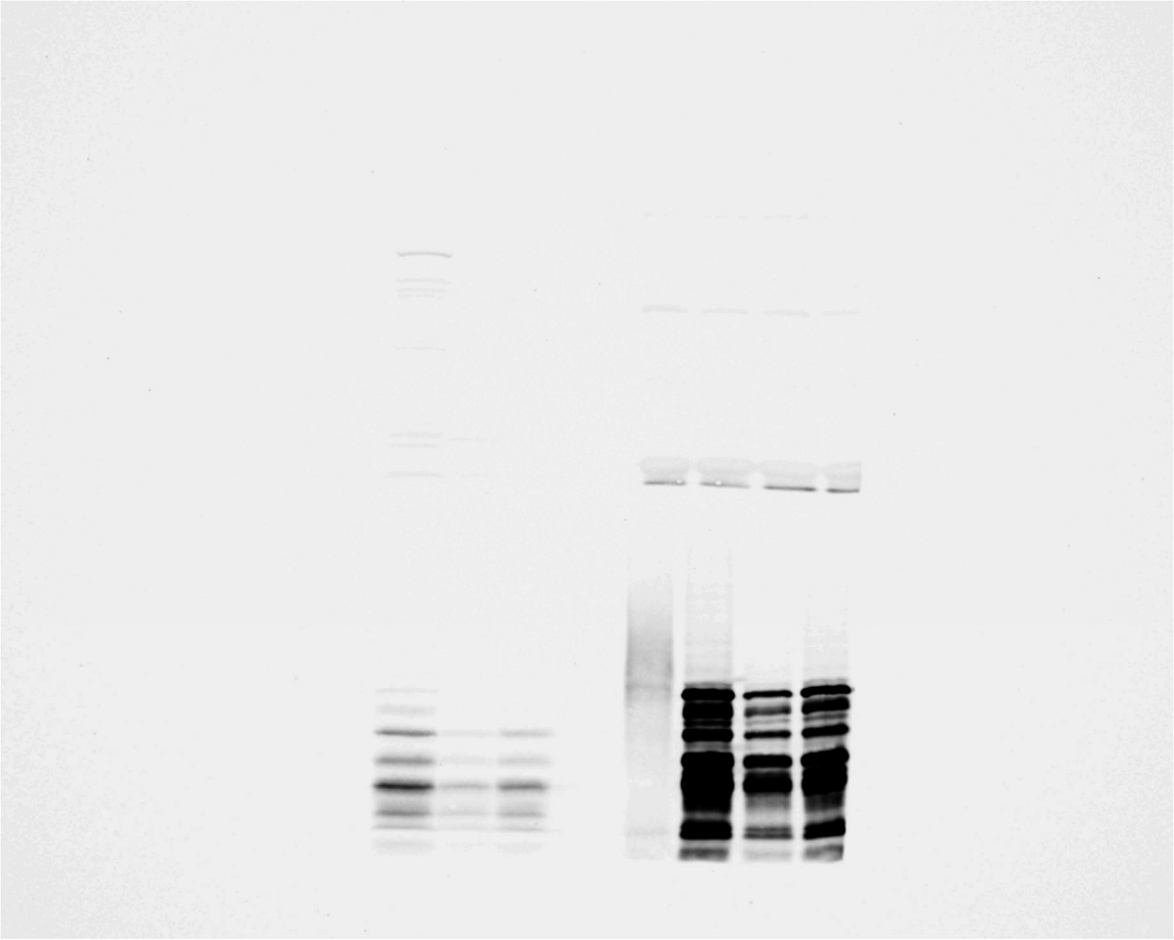

Source Data Supplementary Figure 14B. Anti-HA western blot.

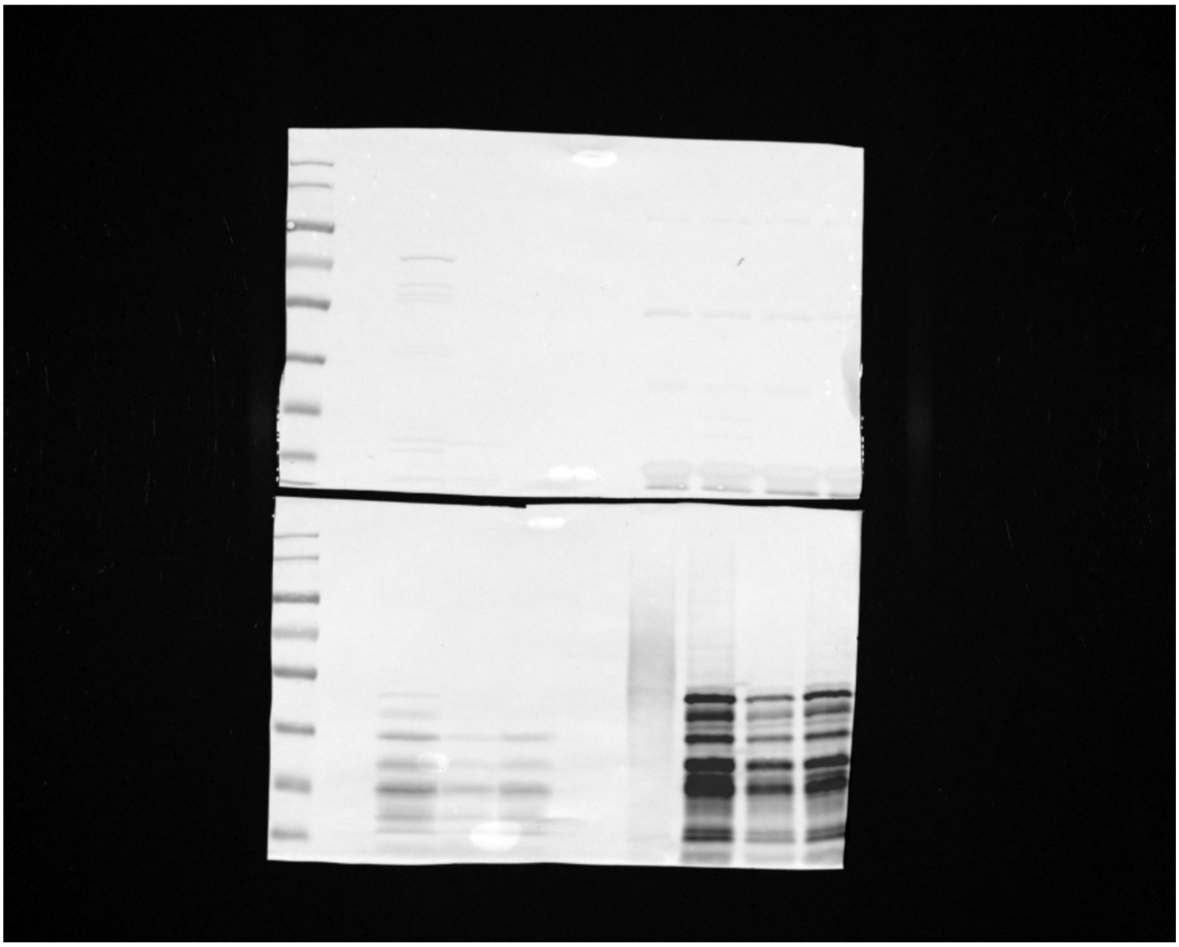

Source Data Supplementary Figure 14B. Anti-HA western blot with molecular weight marker (Left lane). Molecular weight marker: PageRuler prestained protein ladder (Life Technologies, Cat. #26616). Top to bottom (kDa): 180, 130, 100, 70, 55, 40, 35, 25.

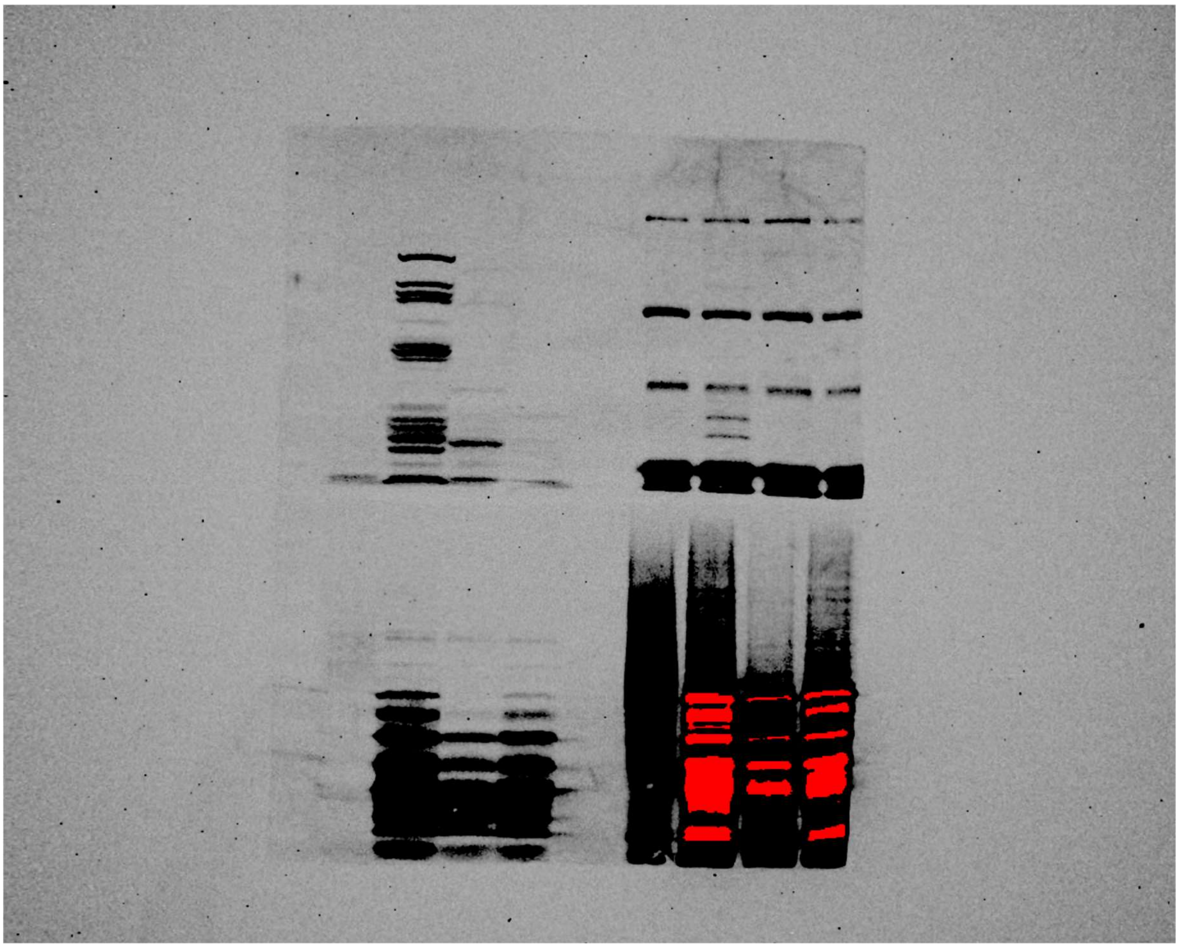

Source Data Supplementary Figure 14B. Anti-Myc western blot.

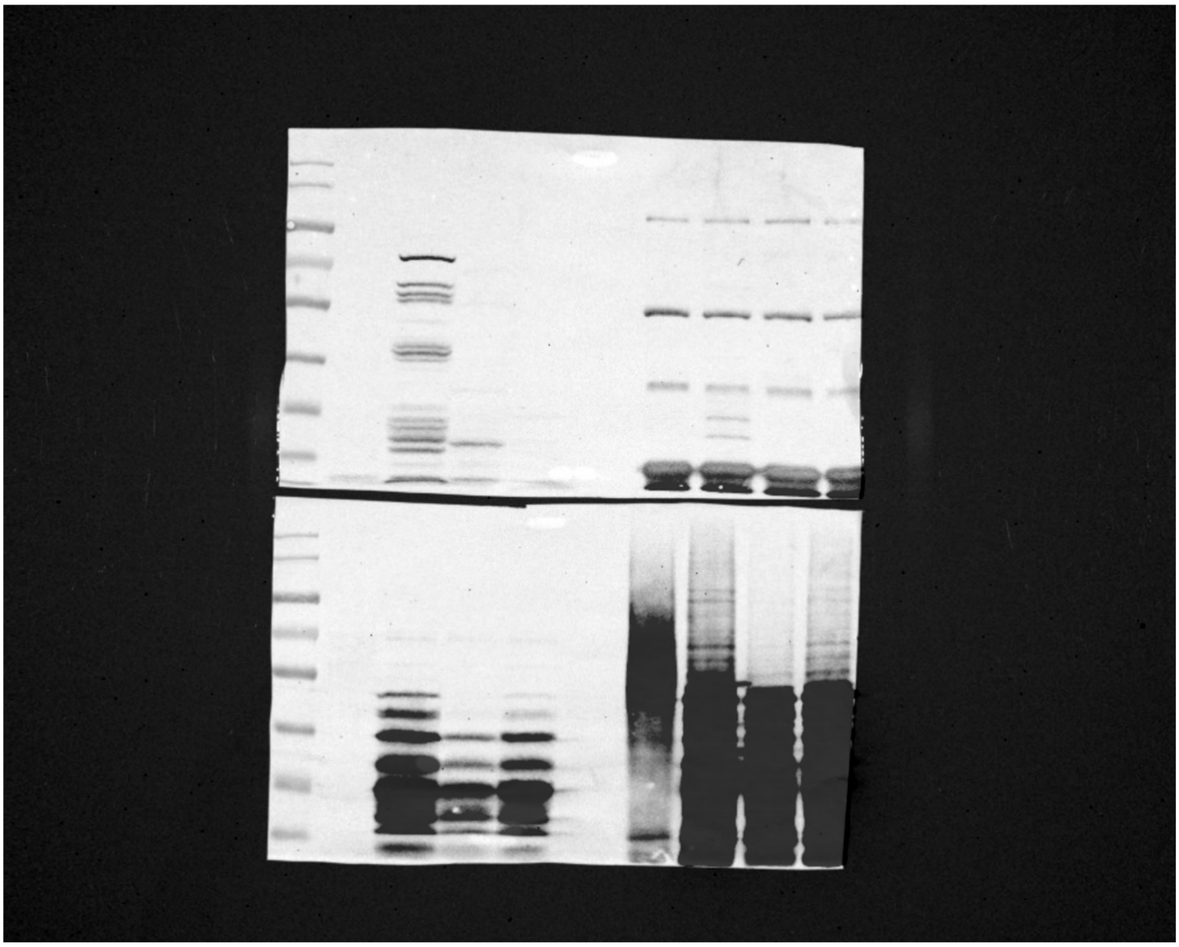

Source Data Supplementary Figure 14B. Anti-Myc western blot with molecular weight marker (left lane). Molecular weight marker: PageRuler prestained protein ladder (Life Technologies, Cat. #26616. Top to bottom (kDa): 180, 130, 100, 70, 55, 40, 35, 25.

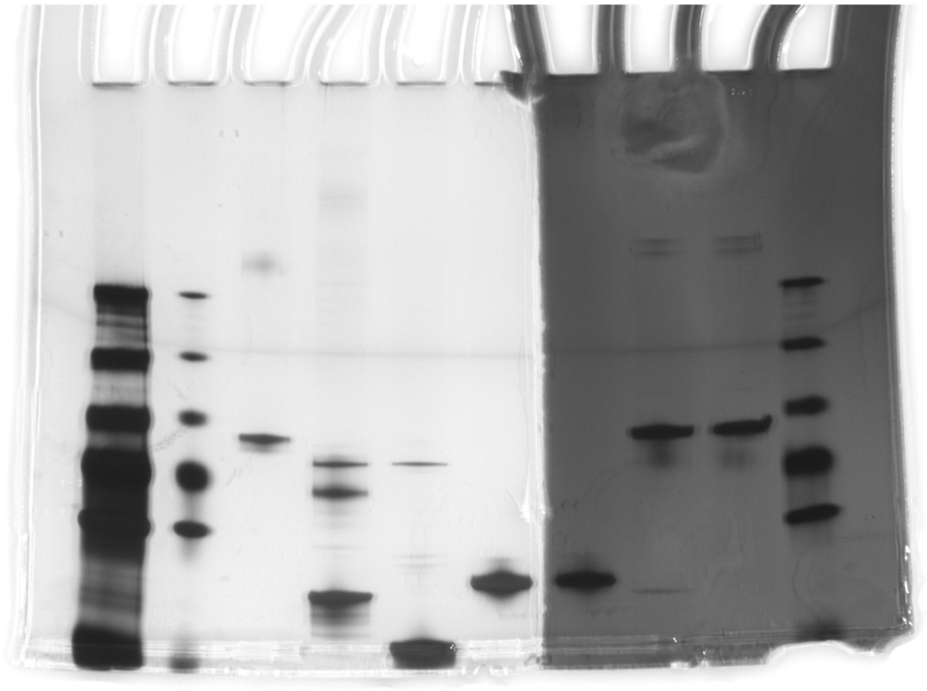

Source Data Supplementary Figure 15.

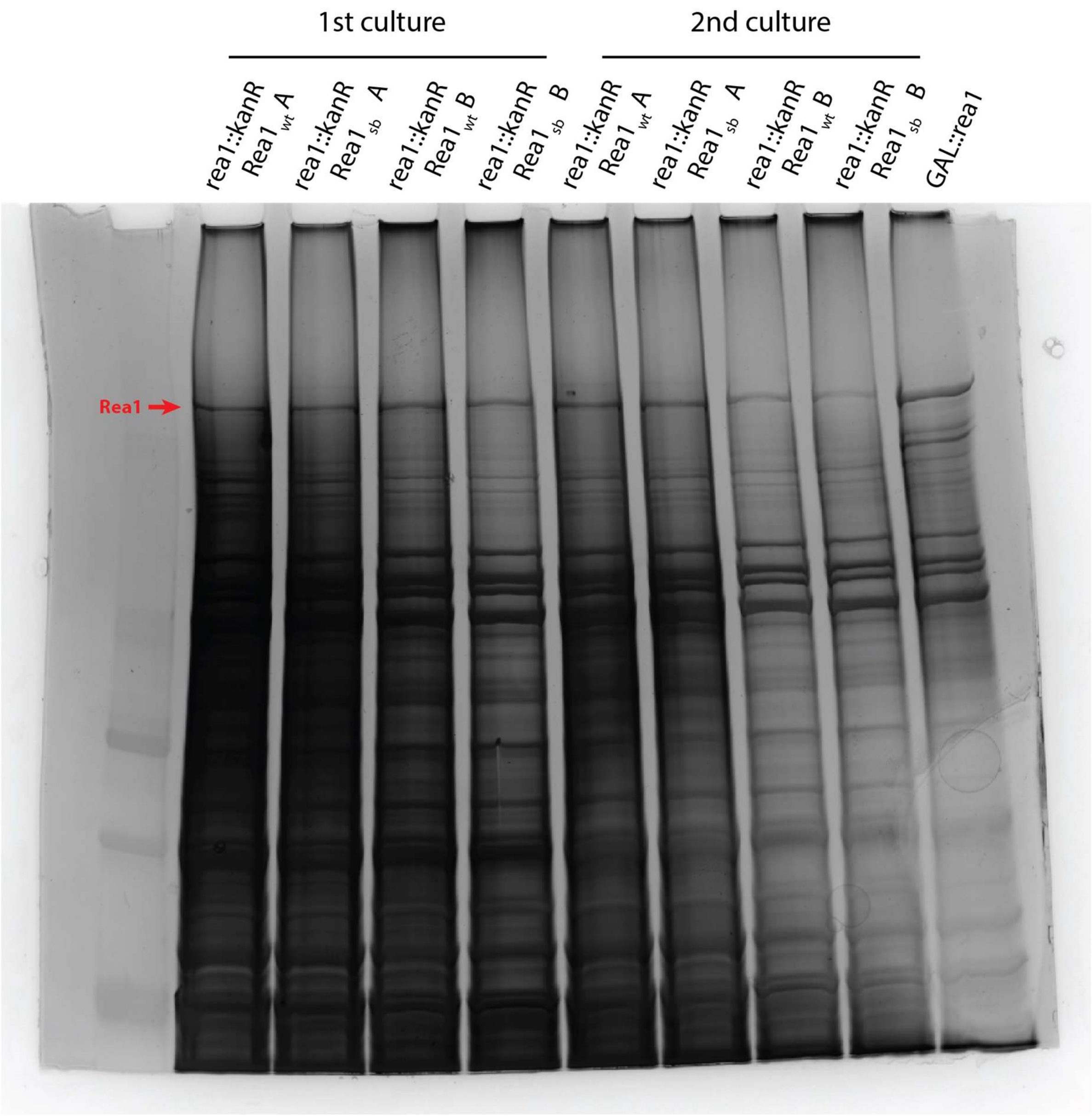

Source Data Supplementary Figure 19.
