# Supplementary material for: Remodelling of Rea1 linker domain drives the removal of assembly factors from pre-ribosomal particles
Source: Nat Commun. 2024 Nov 27;15:10309. doi: 10.1038/s41467-024-54698-w (PMC11603028; doi:10.1038/s41467-024-54698-w)
Supplement: Supplementary file 3 — Description of Additional Supplementary Files [file 41467_2024_54698_MOESM3_ESM.pdf]

Description of Additional Supplementary Files

File name: Supplementary Movie 1

Description: Linker remodelling states 1 - 8 aligned on AAA+ ring.

File name: Supplementary Movie 2

Description: Linker remodelling states 1 - 8 aligned on long linker axis.

File name: Supplementary Movie 3

Description: Microtubule gliding event example 1.

File name: Supplementary Movie 4

Description: Microtubule gliding event example 2.

File name: Supplementary Movie 5

Description: Microtubule gliding event example 3.

File name: Supplementary Movie 6

Description: Linker remodelling in Rea1D2915A-R2976A-D3042A mutant.

File name: Supplementary Movie 7

Description: Microtubule gliding positive control with human dynein 2.
