# Supplementary material for: Remodelling of Rea1 linker domain drives the removal of assembly factors from pre-ribosomal particles
Source: Nat Commun. 2024 Nov 27;15:10309. doi: 10.1038/s41467-024-54698-w (PMC11603028; doi:10.1038/s41467-024-54698-w)
Supplement: Supplementary file 11 — Reporting Summary [file 41467_2024_54698_MOESM11_ESM.pdf]

nature portfolio

Corresponding author(s): Helgo Schmidt

Last updated by author(s): Aug 6, 2024

Reporting Summary

Nature Portfolio wishes to improve the reproducibility of the work that we publish. This form provides structure for consistency and transparency in reporting. For further information on Nature Portfolio policies, see our Editorial Policies and the Editorial Policy Checklist.

Statistics

For all statistical analyses, confirm that the following items are present in the figure legend, table legend, main text, or Methods section.

n/a	Confirmed	
☐	☒	The exact sample size (*n*) for each experimental group/condition, given as a discrete number and unit of measurement
☐	☒	A statement on whether measurements were taken from distinct samples or whether the same sample was measured repeatedly
☒	☐	The statistical test(s) used AND whether they are one- or two-sided<br>*Only common tests should be described solely by name; describe more complex techniques in the Methods section.*
☒	☐	A description of all covariates tested
☒	☐	A description of any assumptions or corrections, such as tests of normality and adjustment for multiple comparisons
☐	☒	A full description of the statistical parameters including central tendency (e.g. means) or other basic estimates (e.g. regression coefficient) AND variation (e.g. standard deviation) or associated estimates of uncertainty (e.g. confidence intervals)
☒	☐	For null hypothesis testing, the test statistic (e.g. *F*, *t*, *r*) with confidence intervals, effect sizes, degrees of freedom and *P* value noted<br>*Give P values as exact values whenever suitable.*
☒	☐	For Bayesian analysis, information on the choice of priors and Markov chain Monte Carlo settings
☒	☐	For hierarchical and complex designs, identification of the appropriate level for tests and full reporting of outcomes
☒	☐	Estimates of effect sizes (e.g. Cohen's *d*, Pearson's *r*), indicating how they were calculated

Our web collection on statistics for biologists contains articles on many of the points above.

Software and code

Policy information about availability of computer code

Data collection	SerialEM version 3-7-14 was used for all electron microscopy data collection
Data analysis	For the cryoEM processing, Relion-3.1 was used. ImageJ version 1.53n was used to analyse the microtubule gliding assays.

For manuscripts utilizing custom algorithms or software that are central to the research but not yet described in published literature, software must be made available to editors and reviewers. We strongly encourage code deposition in a community repository (e.g. GitHub). See the Nature Portfolio guidelines for submitting code & software for further information.

Data

Policy information about availability of data

All manuscripts must include a data availability statement. This statement should provide the following information, where applicable:

- Accession codes, unique identifiers, or web links for publicly available datasets
- A description of any restrictions on data availability
- For clinical datasets or third party data, please ensure that the statement adheres to our policy

The XL-MS dataset has been deposited on the ProteomeXchange Consortium via the PRIDE [41] repository with the dataset identifier PXD053636 and 10.6019/PXD053636 [https://proteomecentral.proteomexchange.org/cgi/GetDataset?ID=PXD053636]. The Mass Photometry raw and treated files generated in this study will be made fully available upon request. CryoEM maps generated in this study have been deposited at the Electron Microscopy Data Bank (EMDB) under accession

codes EMD-50815 [https://www.ebi.ac.uk/emdb/EMD-50815], EMD-50816 [https://www.ebi.ac.uk/emdb/EMD-50816], EMD-50817 [https://www.ebi.ac.uk/emdb/EMD-50817] and EMD-50818 [https://www.ebi.ac.uk/emdb/EMD-50818]. Source data are provided with this paper.

Research involving human participants, their data, or biological material

Policy information about studies with human participants or human data. See also policy information about sex, gender (identity/presentation), and sexual orientation and race, ethnicity and racism.

Reporting on sex and gender	Does not apply to our manuscript
Reporting on race, ethnicity, or other socially relevant groupings	Does not apply to our manuscript
Population characteristics	Does not apply to our manuscript
Recruitment	Does not apply to our manuscript
Ethics oversight	Does not apply to our manuscript

Note that full information on the approval of the study protocol must also be provided in the manuscript.

Field-specific reporting

Please select the one below that is the best fit for your research. If you are not sure, read the appropriate sections before making your selection.

☒ Life sciences ☐ Behavioural & social sciences ☐ Ecological, evolutionary & environmental sciences

For a reference copy of the document with all sections, see nature.com/documents/nr-reporting-summary-flat.pdf

Life sciences study design

All studies must disclose on these points even when the disclosure is negative.

Sample size	We followed the common sample sizes in the relevant literature
Data exclusions	No data was excluded
Replication	We confirm that replications were successful
Randomization	Does not apply to our manuscript, we did not work with patient data
Blinding	Does not apply to our manuscript, we did not work with patient data

Reporting for specific materials, systems and methods

We require information from authors about some types of materials, experimental systems and methods used in many studies. Here, indicate whether each material, system or method listed is relevant to your study. If you are not sure if a list item applies to your research, read the appropriate section before selecting a response.

Materials & experimental systems

n/a	Involved in the study	
☐	☒	Antibodies
☐	☐	Eukaryotic cell lines
☐	☐	Palaeontology and archaeology
☐	☐	Animals and other organisms
☐	☐	Clinical data
☐	☐	Dual use research of concern
☐	☐	Plants

Methods

n/a	Involved in the study	
☐	☐	ChIP-seq
☐	☐	Flow cytometry
☐	☐	MRI-based neuroimaging

Antibodies

Antibodies used	mouse monoclonal anti-HA antibody (Roche Diagnostics, Cat. # 12013819001, 1:1000 dilution), mouse monoclonal anti-Myc antibody (Covalab, ID Covalab: mab20008; Clone 9E10, batch number: 527700, Cat. # 00115009, dilution: 5 µg/ml)

Validation	Anti-HA-Peroxidase, High Affinity (3F10) recognizes the 9-amino acid sequence YPYDVPDYA, derived from the human influenza hemagglutinin (HA) protein.This epitope is also recognized in fusion proteins regardless of its position (N-terminal, C-terminal or internal). The anti-Myc antibody HRP conjugate recognizes the EQKLISEEDL peptide

Eukaryotic cell lines

Policy information about cell lines and Sex and Gender in Research

Cell line source(s)	Not relevant to our manuscript
Authentication	Not relevant to our manuscript
Mycoplasma contamination	Not relevant to our manuscript
Commonly misidentified lines (See ICLAC register)	Not relevant to our manuscript

Palaeontology and Archaeology

Specimen provenance	Not relevant to our manuscript
Specimen deposition	Not relevant to our manuscript
Dating methods	Not relevant to our manuscript

☐ Tick this box to confirm that the raw and calibrated dates are available in the paper or in Supplementary Information.

Ethics oversight	Not relevant to our manuscript

Note that full information on the approval of the study protocol must also be provided in the manuscript.

Animals and other research organisms

Policy information about studies involving animals; ARRIVE guidelines recommended for reporting animal research, and Sex and Gender in Research

Laboratory animals	Not relevant to our manuscript
Wild animals	Not relevant to our manuscript
Reporting on sex	Not relevant to our manuscript
Field-collected samples	Not relevant to our manuscript
Ethics oversight	Not relevant to our manuscript

Note that full information on the approval of the study protocol must also be provided in the manuscript.

Clinical data

Policy information about clinical studies

All manuscripts should comply with the ICMJE guidelines for publication of clinical research and a completed CONSORT checklist must be included with all submissions.

Clinical trial registration	Not relevant to our manuscript
Study protocol	Not relevant to our manuscript
Data collection	Not relevant to our manuscript
Outcomes	Not relevant to our manuscript

Dual use research of concern

Policy information about dual use research of concern

Hazards

Could the accidental, deliberate or reckless misuse of agents or technologies generated in the work, or the application of information presented in the manuscript, pose a threat to:

No	Yes	
☒	☐	Public health
☒	☐	National security
☒	☐	Crops and/or livestock
☒	☐	Ecosystems
☒	☐	Any other significant area

Experiments of concern

Does the work involve any of these experiments of concern:

No	Yes	
☒	☐	Demonstrate how to render a vaccine ineffective
☒	☐	Confer resistance to therapeutically useful antibiotics or antiviral agents
☒	☐	Enhance the virulence of a pathogen or render a nonpathogen virulent
☒	☐	Increase transmissibility of a pathogen
☒	☐	Alter the host range of a pathogen
☒	☐	Enable evasion of diagnostic/detection modalities
☒	☐	Enable the weaponization of a biological agent or toxin
☒	☐	Any other potentially harmful combination of experiments and agents

Plants

Seed stocks	Not relevant to our manuscript
Novel plant genotypes	Not relevant to our manuscript
Authentication	Not relevant to our manuscript

ChIP-seq

Data deposition

☐ Confirm that both raw and final processed data have been deposited in a public database such as GEO.

☐ Confirm that you have deposited or provided access to graph files (e.g. BED files) for the called peaks.

Data access links<br>*May remain private before publication.*	Not relevant to our manuscript
Files in database submission	Not relevant to our manuscript
Genome browser session<br>(e.g. UCSC)	Not relevant to our manuscript

Methodology

Replicates	Not relevant to our manuscript

Sequencing depth	Not relevant to our manuscript
Antibodies	Not relevant to our manuscript
Peak calling parameters	Not relevant to our manuscript
Data quality	Not relevant to our manuscript
Software	Not relevant to our manuscript

Flow Cytometry

Plots

Confirm that:

- [ ] The axis labels state the marker and fluorochrome used (e.g. CD4-FITC).
- [ ] The axis scales are clearly visible. Include numbers along axes only for bottom left plot of group (a 'group' is an analysis of identical markers).
- [ ] All plots are contour plots with outliers or pseudocolor plots.
- [ ] A numerical value for number of cells or percentage (with statistics) is provided.

Methodology

Sample preparation	Not relevant to our manuscript
Instrument	Not relevant to our manuscript
Software	Not relevant to our manuscript
Cell population abundance	Not relevant to our manuscript
Gating strategy	Not relevant to our manuscript

- [ ] Tick this box to confirm that a figure exemplifying the gating strategy is provided in the Supplementary Information.

Magnetic resonance imaging

Experimental design

Design type	Not relevant to our manuscript
Design specifications	Not relevant to our manuscript
Behavioral performance measures	Not relevant to our manuscript

Acquisition

Imaging type(s)	Not relevant to our manuscript
Field strength	Not relevant to our manuscript
Sequence & imaging parameters	Not relevant to our manuscript
Area of acquisition	Not relevant to our manuscript

Diffusion MRI ☐ Used ☒ Not used

Preprocessing

Preprocessing software	Not relevant to our manuscript
Normalization	Not relevant to our manuscript
Normalization template	Not relevant to our manuscript

Noise and artifact removal	Not relevant to our manuscript
Volume censoring	Not relevant to our manuscript

Statistical modeling & inference

Model type and settings	Not relevant to our manuscript
Effect(s) tested	Not relevant to our manuscript

Specify type of analysis: ☐ Whole brain ☐ ROI-based ☐ Both

Statistic type for inference (See Eklund et al. 2016)	Not relevant to our manuscript
Correction	Not relevant to our manuscript

Models & analysis

n/a	Involved in the study
☒	☐ Functional and/or effective connectivity
☒	☐ Graph analysis
☒	☐ Multivariate modeling or predictive analysis
